# Development of novel benzofuran-isatin conjugates as potential antiproliferative agents with apoptosis inducing mechanism in Colon cancer

**DOI:** 10.1080/14756366.2021.1944127

**Published:** 2021-06-28

**Authors:** Wagdy M. Eldehna, Rofaida Salem, Zainab M. Elsayed, Tarfah Al-Warhi, Hamada R. Knany, Rezk R. Ayyad, Thamer Bin Traiki, Maha-Hamadien Abdulla, Rehan Ahmad, Hatem A. Abdel-Aziz, Radwan El-Haggar

**Affiliations:** a Department of Pharmaceutical Chemistry, Faculty of Pharmacy, Kafrelsheikh University, Kafrelsheikh, Egypt; b Scientific Research and Innovation Support Unit, Faculty of Pharmacy, Kafrelsheikh University, Kafrelsheikh, Egypt; c Department of Chemistry, College of Science, Princess Nourah bint Abdulrahman University, Riyadh, Saudi Arabia; d Department of Pharmacognosy, College of Pharmacy, Mansoura University, Mansoura, Egypt; e Department of Pharmaceutical Chemistry, Faculty of Pharmacy, Al-Azhar University, Cairo, Egypt; f Colorectal Research Chair, Department of Surgery, King Khalid University Hospital, King Saud University College of Medicine, Riyadh, Saudi Arabia; g Department of Applied Organic Chemistry, National Research Center, Dokki, Giza, Egypt; h Pharmaceutical Chemistry Department, Faculty of Pharmacy, Helwan University, Cairo, Egypt

**Keywords:** Benzofuran hydrazide, Isatin, Cleaved PARP, Bcl2 inhibitors, Colon cancer

## Abstract

In the current work, a new set of carbohydrazide linked benzofuran-isatin conjugates (**5a–e** and **7a–i**) was designed and synthesised. The anticancer activity for compounds (**5b–d**, **7a, 7b, 7d** and **7g**) was measured against NCI-55 human cancer cell lines. Compound **5d** was the most efficient, and thus subjected to the five-dose screen where it showed excellent broad activity against almost all tested cancer subpanels. Furthermore, all conjugates (**5a–e** and **7a–i**) showed a good anti-proliferative activity towards colorectal cancer SW-620 and HT-29 cell lines, with an excellent inhibitory effect for compounds **5a** and **5d** (IC_50_ = 8.7 and 9.4 µM (**5a**), and 6.5 and 9.8 µM for (**5d**), respectively). Both compounds displayed selective cytotoxicity with good safety profile. In addition, both compounds provoked apoptosis in a dose dependent manner in SW-620 cells. Also, they significantly inhibited the anti-apoptotic Bcl2 protein expression and increased the cleaved PARP level that resulted in SW-620 cells apoptosis.

## Graphical Abstract

**Figure:**
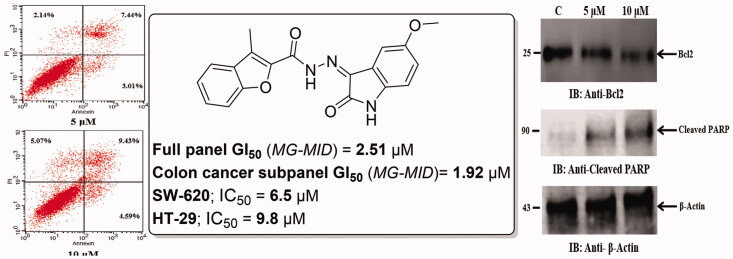

## 1. Introduction

Cancer, a large family of diseases, is characterised by fast and uncontrolled cell division and differentiation mechanisms and has the potential to spread to or invade other body parts^1^. For several decades, cancer is considered one of the major world public health problems, and it remains a serious reason of the death of human beings all over the world^2^. Despite the presence of a variety of cancer treatment strategies, the majority of which induces non-selective cell death by targeting the DNA synthesis^3–6^ and/or the replication machinery^7–10^. These early strategies are accompanied by severe side effects due to the unspecific cytotoxicity towards the cancer cells in addition to the resistance developed against them^4^. Therefore, the development of safe and effective novel anticancer agents with increased selective treatment strategies towards cancer cells has received more attention and still ongoing active search^11^^,^^12^.

Recent strategies for anticancer development are to target specific biomarkers required for cancer cells division and/or induction of cell apoptosis such as deregulated, mutated, or over expressed proteins^13^ and thus, affect cancer cells selectively with minimum influences on normal cells^14^. Among these targets are the anti-apoptotic protein Bcl2 and Poly ADP-ribose polymerase (PARP). In this regard, several reports stated that numerous of cancer cells are characterised by anti-apoptotic proteins (Bcl2) over-expression, which could lead to prevention of cell apoptosis as well as development of drug resistance^15^^,^^16^. On the other hand, PARP is a family of proteins involved in numerous cellular functions such as DNA repair and genomic stability^17–19^ and also, PARP was reported to activate programmed cell death, through cleavage into PAR (Poly ADP-ribose), which motivates mitochondria to produce apoptosis inducing factors^20^. Thus, the development of compounds that inhibit the antiapoptotic Bcl2 proteins and/or potentiate the cleavage of PARP could be a promising approach to identify new anticancer therapies.

Heterocyclic compounds in particular oxygen containing heterocycles represent an important class of compounds possessing interesting pharmacological and biological activities^21–23^. Benzofuran nucleus, as a key functional scaffold, represents a basic structure in a diversity of biologically active synthetic and natural products^24–26^, with broad range of desirable activities including; anti-Alzheimer’s^27^, antibacterial^28^, anti-tubercular^29^, antioxidant^30^, anti-inflammatory^31^, as well as antitumor activities^32^. Benzofuran derivatives exert their antiproliferative activity with diversified mechanisms such as inhibition of tubulin polymerisation^33^^,^^34^, HIF-1^35^, Aurora B kinase^36^ and VEGFR-2 activity^37^. Furthermore, some benzofurans mediate their antiproliferative activity via apoptosis induction in various human cancer cell lines^38–40^. In addition, benzofuran-based conjugates were largely studied and were found to exert significant anticancer activity, such as conjugation of benzofuran with pyrazole^41^, indole^42^ and others^43^^,^^44^.

**Figure 1.**
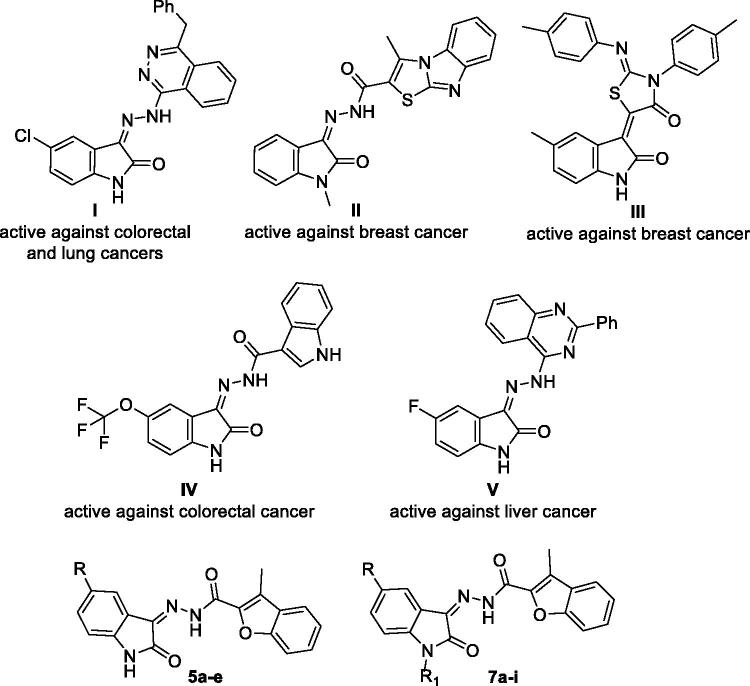
Structures of some reported isatin-bearing conjugates (**I–V**), as well as structures for target benzofuran-isatin conjugates (**5a–e** and **7a–i**).

On the other hand, isatin is identified as a privileged nucleus that included in many pharmacologically active small molecules, such as antiviral^45^, antimicrobial^46^, anticonvulsant^47^, CNS-acting^48^, as well as anticancer^49^^,^^50^ agents. Over the last few years, hybridisation of isatin nucleus with different heterocycles has been reported as a successful approach to develop efficient antitumor agents towards different cancer types through diverse enzymatic and cellular mechanisms^49^^,^^50^. To name just a few, isatin-phthalazine (compound **I**)^51^, isatin-thiazolo[3,2-*a*]benzimidazole (compound **II**)^52^, isatin-thiazolidinone (compound **III**)^53^, isatin-indole (compound **IV**)^54^ and isatin-quinazoline (compound **V**)^55^ conjugates were reported to possess promising anticancer activities (Figure 1).

Encouraged by the aforementioned findings and considering the need to develop safe and effective novel anticancer agents, a new attempt to study the significance of utilisation of heterocycles hybridisation approach to furnish efficient anti-proliferative activity was reported herein. A novel series of benzofuran-isatin conjugates (**5a–e** and **7a–i**, Figure 1) linked by a carbohydrazide group, was designed and synthesised. The new compounds were screened for their potential anticancer activity following NCI, USA protocol against fifty-five different cell lines under nine different cancer panels. In addition, the cytotoxic effect of these conjugates against SW-620 and HT-29 colorectal cancer cell lines was investigated and their ability to induce cell apoptosis was examined. Furthermore, the level of the mitochondrial antiapoptotic protein Bcl2 and the level of cleaved PARP in both SW-620 and HT-29 colorectal cancer cell lines were also determined.

## 2. Results and discussion

### 2.1. Chemistry

**Scheme 1.**
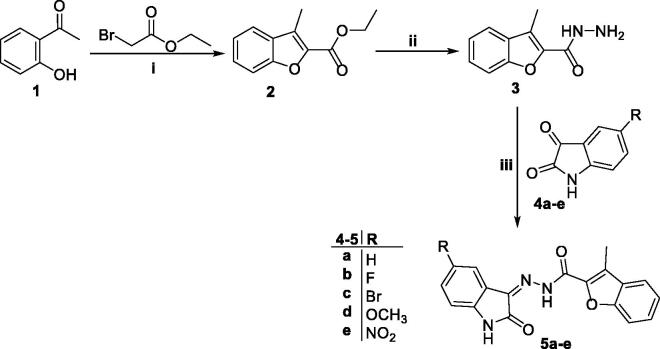
Synthesis of target conjugates **5a–e**; (i) Anhydrous CH_3_CN/potassium carbonate/reflux 8 h, (ii) Hydrazine hydrate/methanol/reflux 4 h, (iii) Ethanol/drops glacial acetic acid (Cat.)/reflux 3–6 h.

The adopted synthetic strategy to develop the target *N*-unsubstituted 3-methyl-*N*′-(oxoindolin-3-ylidene)benzofuran-2-carbohydrazide derivatives **5a–e** was outlined in Scheme 1.

Key starting ester 3-methylbenzofuran-2-carboxylate **2**, was prepared in 85% yield through cyclisation of 1–(2-hydroxyphenyl)ethan-1-one **1** and ethyl bromoacetate in anhydrous acetonitrile with the presence of potassium carbonate. Thereafter, heating of ester derivative **2** with hydrazine hydrate in methanol afforded the corresponding key intermediate 3-methylbenzofuran-2-carbohydrazide **3**. Finally, carbohydrazide **3** was condensed with different indoline-2,3-dione derivatives **4a–e**, through heating under reflux temperature in absolute ethyl alcohol and few drops of acetic acid, to give the desired benzofuran-based compounds **5a–e**, respectively, in 72–89%yield.

**Scheme 2.**
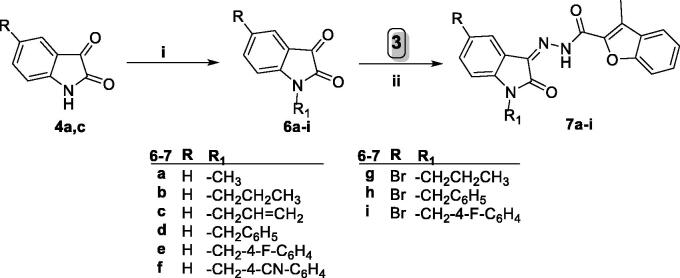
Synthesis of target benzofurans **7a–i**; (i) (R-Br or Ar-Br)/Acetonitrile/KI (Cat.)/potassium carbonate/reflux 3 h, (ii) Ethanol absolute/drops glacial acetic acid (Cat.)/reflux 3–6 h.

On the other hand, Scheme 2 illustrated the synthetic pathway utilised to synthesise *N*-substituted 3-methyl-*N*′-(oxoindolin-3-ylidene)benzofuran-2-carbohydrazide derivatives **7a–i**. In this scheme, alkylation of indoline-2,3-diones **4a** and **4c** was accomplished *via* heating with different alkyl bromide or benzyl bromide derivatives in anhydrous acetonitrile to produce *N*-substituted indoline-2,3-dione derivatives **6a–i**. Then indoline-2,3-diones **6a–i** were condensed with the key intermediate carbohydrazide **3** producing target benzofurans **7a–i**, respectively, in 75–87% yield.

Structures of the newly prepared benzofuran-based derivatives **5a–e** and **7a–i** were verified based on spectral and elemental analyses. ^1^H NMR spectra of **5a–e** and **7a–i** revealed the presence of two singlet peaks for the protons of C-3 CH_3_ of benzofuran ring and NH of the hydrazide linker at range *δ* (2.52–2.72) and (11.37–14.10) *ppm,* respectively. Moreover, structure of compounds **5a–e** was confirmed *via* presence of another singlet D_2_O exchangeable signal attributable to the proton of NH for isatin moieties at *δ* 10.91–11.98 *ppm*. In addition, ^1^H NMR spectra of *N*-benzyl bearing derivatives **7d–f**, **7h** and **7i** displayed the characteristic singlet signal of the benzylic protons at *δ* 4.98–5.07 *ppm*, while spectra for hybrids **7a**, **7b** and **7g** revealed the presence of the aliphatic protons corresponding to the *N*-substituents in these derivatives at *δ* (3.28 *ppm*), (0.93, 1.66 and 3.76 *ppm*) and (0.97, 1.69 and 3.80 *ppm*), respectively.

On the other hand, ^13 ^C NMR spectra for the novel compounds **5a–e** and **7a–i** showed one signal belonging to the carbon of CH_3_ of benzofuran ring at *δ* 8.12–9.49 *ppm*, also, they showed two signals belonging to the carbon of C = O functionalities for both the hydrazide linker and isatin moiety at range *δ* (161.15–163.62) and (164.08–167.02) *ppm*, respectively. In addition, the existence of benzylic carbon in *N*-benzyl bearing derivatives **7d–f**, **7h** and **7i** was confirmed by a signal at *δ* 42.14–46.03 *ppm*, whereas, the carbons of propyl moiety in compounds **7b** and **7g** appeared as signals at range *δ* (11.68–13.00), (20.92–22.98) and (41.30–48.74) *ppm*.

### 2.2. Biological evaluation

#### 2.2.1. Nci screening of anticancer activity

In the present investigation, the chemical structures for the novel benzofuran-isatin conjugates were presented to the Developmental Therapeutics Program at the National Cancer Institute (NCI), USA. Seven conjugates (**5b–d**,**7a, 7b, 7d** and **7g**) were selected, according to NCI’s-DTP selection guidelines^56^, for evaluating their potential *in vitro* anticancer activity against a panel of fifty-five human cancer cell lines representing nine tumour panels according to the NCI, Bethesda, Drug Evaluation Branch protocol^57^^,^^58^.

##### 2.2.1.1. Preliminary single high dose screening at 10 μM concentration

**Figure 2.**
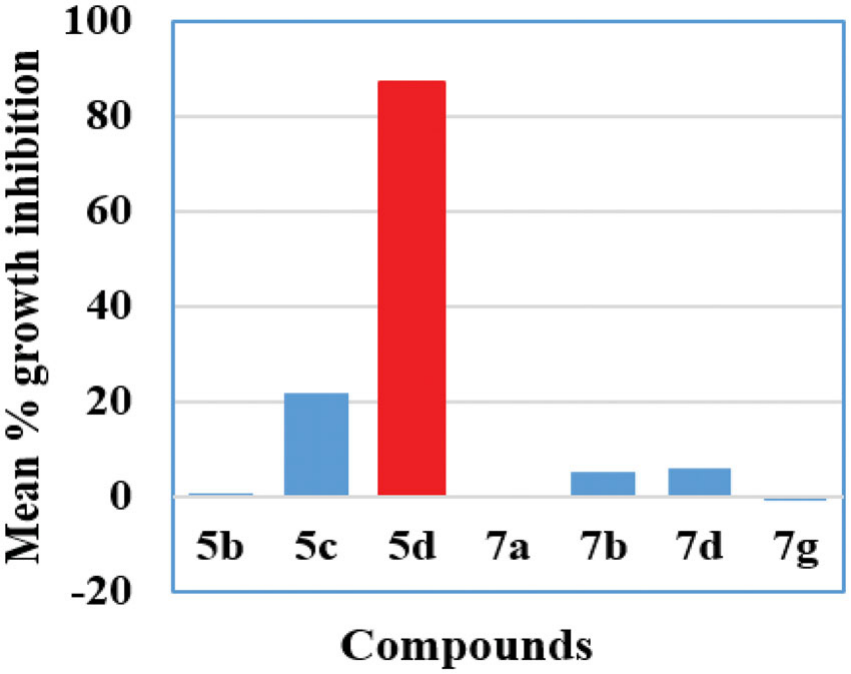
Mean % growth inhibition of compounds **5b–d**,**7a, 7b, 7d** and **7g** against NCI-55 cancer cell line panel.

Firstly, the seven selected conjugates (**5b–d**,**7a, 7b, 7d** and **7g**) were screened at a dose of 10 μM for their antiproliferative activity against a panel of fifty-five cancer cell lines. The mean percent growth inhibition values (GI%) for conjugates **5b–d**,**7a, 7b, 7d** and **7g** against NCI-55 cancer cell lines were displayed in (Figure 2, Table 1**)**. The primary assay data analysis revealed that the new benzofuran-isatin hybrids showed weak to moderate inhibitory activity some of the subpanel cancer cell lines except for compound **5d** that possessed excellent activity against nearly all the cancer cell lines. Although compound **5b**, **7a**, **7b**, **7d** and **7g** proved inactive against most of the subpanels cancer cell lines with mean GI% = 0.75%, 0.09%, 5.18%, 6.01%, and −0.8%, respectively, they showed selective moderate anticancer activity against certain cell lines such as Ovarian-IGROV1, Non-small cell lung-EKVX, Renal-UO-31 and Breast/MCF7 cancer cell lines with GI% range 17–53% (Table 1).

**Table 1.** In vitro Anticancer screening results of compounds **5b–d**,**7a, 7b, 7d** and **7g** against fifty-five human tumour cell lines with single dose assay (10^−5^ M concentration). Data was provided as cell growth inhibition percentage.

Subpanel / tumour cell lines	Compound^a^						
	5b	5c	5d	7a	7b	7d	7g
Leukaemia							
CCRF-CEM	–	–	42.92	–	–	–	–
MOLT-4	–	–	49.14	–	–	–	–
HL-60(TB)	–	21.46	46.13	–	10.94	–	–
K-562	–	18.04	57.50	–	11.45	–	–
SR	–	17.62	56.52	–	–	–	–
RPMI-8226	–	–	NA	NT	NT	–	NT
Non-small cell lung cancer							
EKVX	29.63	32.13	62.73	25.07	32.35	30.23	18.10
A549/ATCC	–	27.92	123.59	–	–	–	–
HOP-92	–	26.54	143.99	–	–	12.64	–
HOP-62	–	41.41	124.09	–	18.06	20.82	–
NCI-H322M	–	31.80	69.41	–	11.87	17.83	–
NCI-H23	–	33.05	89.35	–	15.06	13.19	–
NCI-H522	10.36	10.33	45.72	–	10.32	21.72	–
NCI-H460	–	42.60	122.83	–	15.60	24.98	–
Colon cancer							
HCC-2998	–	–	56.46	–	–	–	–
COLO 205	–	–	77.24	–	–	–	–
SW-620	–	10.57	64.85	30.57	–	11.21	–
HCT-116	–	32.03	128.55	–	–	–	–
HCT-15	–	–	50.91	–	–	–	–
HT-29	–	–	72.67	–	–	–	–
KM12	–	20.46	56.20	–	–	–	–
CNS cancer							
SF-539	–	57.88	62.58	–	–	–	–
SF-268	–	11.66	42.33	–	–	–	–
SF-295	–	20.30	94.95	11.84	11.86	–	–
U251	–	42.58	97.95	–	–	–	–
SNB-19	–	31.48	57.29	–	–	11.60	–
Melanoma							
MALME-3M	12.43	64.93	129.46	–	–	–	16.04
LOX IMVI	–	33.82	83.71	–	–	13.84	–
MDA-MB-435	–	–	61.04	–	–	–	–
M14	–	25.40	71.32	–	–	–	–
UACC-257	–	–	49.98	–	–	–	–
UACC-62	10.91	30.94	65.68	–	16.86	20.56	–
SK-MEL-2	–	–	29.13	–	–	–	–
SK-MEL-28	–	–	84.16	–	–	–	–
Ovarian cancer							
NCI/ADR-RES	–	29.33	69.51	–	10.33	10.19	–
IGROV1	33.72	44.59	93.67	32.80	42.55	33.59	22.38
OVCAR-3	–	–	165.51	–	–	–	–
OVCAR-8	–	22.46	62.23	–	–	–	–
OVCAR-4	–	31.04	15,285	–	–	–	–
OVCAR-5	–	–	52.33	–	–	–	–
Renal cancer							
786-0	–	34.70	166.85	–	–	–	–
CAKI-1	19.50	26.59	151.66	19.44	11.31	18.98	14.14
ACHN	–	35.48	150.93	–	11.60	13.30	–
SN12C	–	29.81	65.13	–	–	–	–
RXF 393	–	11.08	136.15	–	–	–	–
UO-31	26.72	43.42	155.33	–	34.27	33.64	21.86
TK-10	–	–	193.95	–	–	–	–
Prostate cancer							
PC-3	–	13.75	78.23	–	10.82	15.73	–
DU-145	–	17.00	82.38	–	–	–	–
Breast cancer							
MCF7	19.04	26.66	77.14	28.25	25.04	16.66	13.20
BT-549	–	22.06	29.88	–	–	–	–
MDA-MB-231/ATCC	14.64	29.88	76.19	–	28.99	18.32	–
HS 578 T	–	46.27	74.50	–	–	–	–
MDA-MB-468	–	–	80.10	–	–	–	–
T-47D	–	42.74	139.16	21.80	23.76	33.31	–
Mean inhibition, %	0.75	21.99	87.33	0.09	5.18	6.01	–0.8
Sensitive cell lines no.	9	40	54	7	19	20	6

^a^ Only GI % higher than 10% are shown. NT: not tested.

In particular, compound **5d** was the most efficient anti-proliferative agent and exhibited excellent activity against almost all subpanel cancer cell lines with mean growth inhibitory activity of 87.33%. Remarkably, compound **5d** exerted excellent growth inhibition properties against Non-small cell lung cancer (NCI-H23), CNS cancer (SF-295, U251), Melanoma (LOX IMVI, SK-MEL-28), Ovarian cancer (IGROV1), Prostate cancer (DU-145) and Breast cancer (MDA-MB-468) cell lines with GI% of 89.35, 94.95, 97.95, 83.71, 84.16, 93.67, 82.38 and 80.10%, respectively (Table 1). In addition, conjugate **5d** showed good potency with GI% equals or greater than 60% towards Non-small cell lung cancer (EKVX), Colon cancer (COLO 205, HT-29 and SW-620), Melanoma (M14, MDA-MB-435 and UACC-62), Ovarian cancer (OVCAR-8), CNS cancer (SF-539), Renal cancer (SN12C), Breast cancer (MCF7, MDA-MB-231/ATCC and HS 578 T) and Prostate cancer (PC-3) cell lines with GI% of 62.73, 77.24, 72.67, 64.85, 71.32, 61.04, 65.68, 62.23, 62.58, 65.13, 77.14, 76.19, 74.50and 78.23% respectivly (Table 1).

It is worthy to mention that **5d** exhibited a lethal cytotoxic impact with GI% >100 against Non-small cell lung cancer (HOP-62, A549/ATCC, HOP-92 and NCI-H460), Colon cancer (HCT-116), Melanoma (MALME-3M), Ovarian cancer (OVCAR-4 and OVCAR-3), Renal cancer (CAKI-1, 786–0, RXF 393, ACHN, TK-10 and UO-31) and Breast cancer (T-47D) cells with GI% values equals 124.09, 123.59, 143.99, 122.83, 128.55, 129.46, 152.85, 165.51, 151.66, 166.85, 136.15, 150.93, 193.95, 155.33 and 139.16%, respectively (Table 1).

On the other hand, compound **5c** showed moderate to good activity against some cell lines with mean GI% = 21.99%. The best results of compound **5c** was against cancer cell lines Non-small cell lung-HOP-62 (GI% = 41.41%), Non-small cell lung-NCI-H460 (GI% = 42.60%), Renal-UO-31 (GI% = 43.42%), Ovarian-IGROV1 (GI% = 44.59%), Breast-HS-578T (GI% = 46.27%), CNS-SF-539 (GI% = 57.88%) and Melanoma-MALME-3M (GI% = 64.93%) (Table 1).

##### 2.2.1.2. In vitro *5 dose full NCI-55 cell panel screening.*

The preliminary screening results showed that conjugate **5d** (NSC: D-819833/1) was the most potent compound in the present study, and displayed effectiveness towards various cell lines represent numerous tumour subpanels (Figure 2). Accordingly, **5d** was promoted to the five-dose (0.01–100 µM) screening assay. Accordingly, three main response parameters (GI_50_, TGI and LC_50_) towards each of the examined cancer cell line were calculated for hybrid **5d** and displayed in Table 2. Where, GI_50_ values represents molar concentration which produces 50% inhibitory effect in the net cell growth; TGI (cytostatic activity) is the molar concentration with total growth inhibition and LC_50_ is the cytotoxicity parameter that reflects the molar concentration that results in 50% net cell death. In addition, the mean graph midpoints (MG-MID), representing the GI_50_ average for the individual subpanels as well as the full panel cell lines were calculated giving an average potency parameter for the examined compound **5d**, (Table 3). Furthermore, by dividing the full panel MID by their individual subpanel MID, the selectivity index of compound **5d** was calculated and was used to measure the selectivity of **5d** towards different cancer cell subpanels.

**Table 2.** NCI *in vitro* screening results (GI_50_, TGI, and LC_50_ (μM) of **5d** (NSC: D-819833/1) in the five-dose test.

Subpanel /tumour cell lines	Compound **5d**		
	GI_50_(µM)	TGI(µM)	LC_50_(µM)
Leukaemia			
CCRF-CEM	NT	>100	>100
HL60(TB)	>100	>100	>100
K-562	NT	>100	>100
MOLT-4	NT	>100	>100
SR	NT	>100	>100
Non-small cell lung cancer			
A549/ATCC	NT	NT	NT
EKVX	2.94	>100	>100
HOP-62	1.92	4.02	8.44
HOP-92	1.84	3.98	NT
NCI-H226	2.19	NT	>100
NCI-H23	1.86	4.43	>100
NCI-H322M	NT	NT	>100
NCI-H460	NT	NT	NT
NCI-H522	6.07	>100	>100
Colon cancer			
COLO 205	NT	>100	>100
HCC-2998	NT	>100	>100
HCT-116	1.92	NT	NT
HCT-15	NT	>100	>100
HT29	NT	NT	>100
KM12	NT	>100	>100
SW-620	NT	>100	>100
CNS cancer			
SF-268	5.18	56.8	>100
SF-295	2.03	4.13	NT
SF-539	1.66	3.16	NT
SNB-19	3.45	16.2	>100
SNB-75	1.25	2.75	6.04
U251	2.03	4.26	NT
Melanoma			
LOX IMVI	3.16	>100	>100
MALME-3M	1.81	3.79	NT
M14	NT	>100	>100
MDA-MB-435	NT	>100	>100
SK-MEL-2	2.56	6.62	54.7
SK-MEL-28	NT	NT	NT
SK-MEL-5	NT	NT	>100
UACC-257	NT	>100	>100
UACC-62	5.19	>100	>100
Ovarian cancer			
IGROV1	2.10	NT	>100
OVCAR-3	1.84	NT	NT
OVCAR-4	NT	NT	NT
OVCAR-5	NT	NT	>100
OVCAR-8	3.22	>100	>100
NCI/ADR-RES	2.47	NT	>100
SK-OV-3	1.82	3.83	NT
Renal cancer			
786-0	1.99	3.84	NT
A498	1.63	4.04	NT
ACHN	1.77	NT	NT
CAKI-1	1.56	3.24	NT
RXF 393	1.79	3.70	NT
SN12C	2.82	>100	>100
TK-10	2.32	4.02	NT
UO-31	NT	NT	NT
Prostate cancer			
PC-3	NT	>100	>100
DU-145	NT	NT	>100
Breast cancer			
MCF7	NT	>100	>100
MDA-MB-231/ATCC	2.07	5.10	>100
HS 578 T	2.41	7.72	>100
BT-549	5.38	33.7	>100
T-47D	1.70	NT	>100
MDA-MB-468	2.60	6.52	>100

NT: not tested.

**Table 3.** Median growth inhibitory concentrations^a^ (GI_50_, µM) of *in-vitro* cancer cell lines subpanel for compound **5d**.

Subpanel /tumour cell lines	Compound **5d**	
	MG-MID	Selectivity index
non-small cell lung cancer	2.80	0.89
Colon Cancer	1.92	1.30
CNS Cancer	2.60	0.96
Melanoma	3.18	0.78
Ovarian Cancer	2.29	1.09
Renal Cancer	1.98	1.26
Breast Cancer	2.83	0.88
Full panel MG-MID^b^	2.51	

^a^ Median value assessed according to the results obtained from NCI’s screening.

^b^ GI_50_ (µM) full panel mean-graph midpoint (MG-MID) = the average sensitivity for all cell lines towards the examined compound.

Results displayed in Table 2, revealed that conjugate **5d** exhibited powerful anti-proliferative activity at a single-digit micromolar level towards all the examined human cancer cell subpanels with GI_50_ values range: 1.25 − 6.07 µM, except for Melanoma HL60(TB) cell line (more than100 µM). Moreover, regarding the cytostatic activity, hybrid **5d** exhibited excellent cytostatic activity with TGI values range 2.75–7.72 µM against numerous cell lines including NSCLC (HOP-62, NCI-H23 and HOP-92), CNS Cancer (SF-295, SF-539, SNB-75 and U251), Melanoma (MALME-3Mand SK-MEL-2), Ovarian Cancer (OVCAR-8), Renal Cancer (786–0, A498, CAKI-1, RXF 393 andTK-10) and Breast Cancer **(**MDA-MB-231/ATCC, HS 578 T and MDA-MB-468). On the other hand, while, compound **5d** showed weak to moderate cytostatic activity towards CNS Cancer **(**SF-268 and SNB-19), and Breast Cancer **(**BT-549) with TGI = 56.8, 16.2 and 33.7 µM, respectively, it proved to have no cytostatic impact (TGI >100 µM) against entire Leukaemia, Colon cancer and Prostate Cancer and the remaining examined cancer cell lines (Table 2). Furthermore, compound **5d** as revealed by the results could be considered as a non-lethal agent that exhibited LC_50_ values more than 100 µM for the all of cancer cell lines herein examined, except for three cancer cell lines; Non-Small Cell Lung Cancer (HOP-62), CNS Cancer **(**SNB-75) and Melanoma (MASK-MEL-2) which possessed a lethal effect of IC_50_ = 8.44, 6.04 and 54.7, respectively (Table 2).

On the other hand, as shown in Table 3, all tested subpanels were sensitive to compound **5d** with MG-MID spinning between 1.92 and 3.18 µM and the most susceptible subpanels were Colon Cancer and Renal Cancer that exhibited MG-MID = 1.92 and 1.98 µM, respectively. Furthermore, it is well known that compounds with selectivity index between 3 and 6 are considered to be of a moderate selectivity, ratios more than six indicated high selectivity towards the corresponding cell line, while compounds not meeting either of these values are considered as non-selective^59^. Therefore, as displayed in the Table 3, the calculated selectivity index for compound **5d** ranged from 0.78 to 1.30 indicated that conjugate **5d** has non-selective, broad spectrum antiproliferative activity against all tested subpanels cancer cells.

#### 2.2.2. In vitro *anti-cancer activity against SW-620 and HT-29colorectal cancer cell lines*

**Figure 3.**
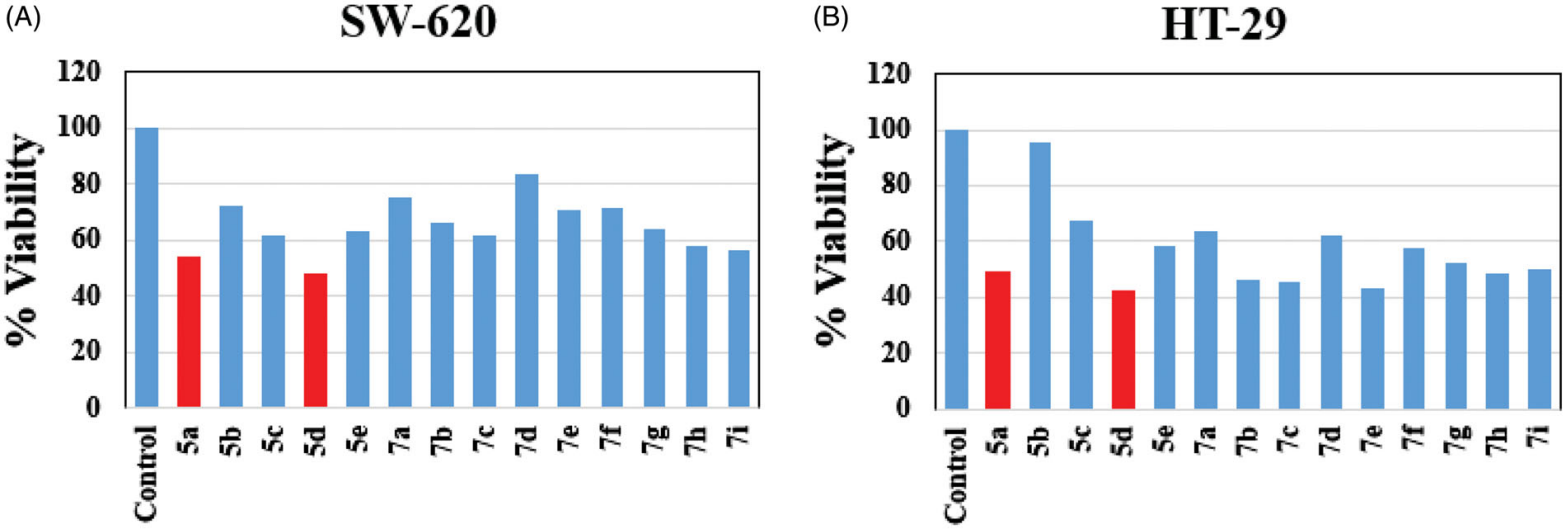
Effect of benzofuran–isatin conjugates (**5a–e** and **7a–i**) on the cell viability. (A) SW-620 with hybrids **5a–e** and **7a–i**, (B) HT-29 with hybrids **5a–e** and **7a–i**.

In the present investigation a new set of benzofuran–isatin hybrids (**5a–e** and **7a–i**) was synthesised to be evaluated for their potential anticancer activity towards two human colorectal cancer cell lines, SW-620 and HT-29. The anticancer activity of the new conjugates was assessed using MTT assay^60^, and the results were shown in Figure 3. The most active compound in the NCI assay (**5d**), in addition to another one from untested compounds by NCI (**5a**), were selected to explore their activity. Both, SW-620 and HT-29 cells were treated with 10 µM of each compound for 24 h and the percent cell viability was calculated using MTT assay. Regarding impact of the target conjugates towards SW-620 cancer cells viability, compound **5d** exhibited about 52% inhibition, whereas, compound **5a** showed 46% inhibition. On the other hand, the results showed that seven compounds (**5a**, **5d**, **7b**, **7c**, **7e**, **7h** and **7i**) showed >50% inhibition of HT-29 cancer cells viability **(**Figure 3**)**.

**Figure 4.**
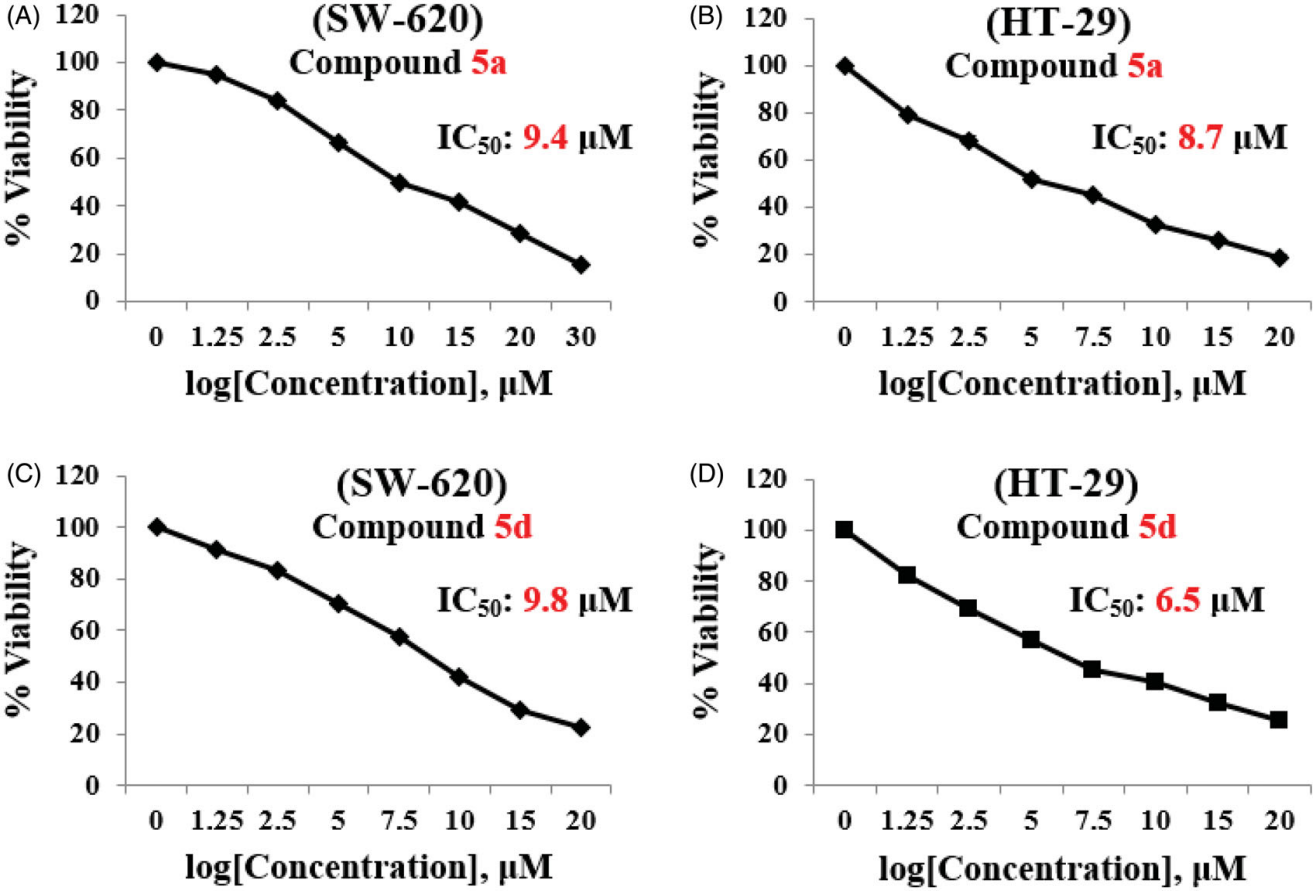
IC_50_ of Compound **5a** and **5d**. (A) SW-620 with compound **5a**, (B) HT-29 with **5a**, (C) SW-620 with compound **5d**, and (D) HT-29 with **5d**.

The results revealed that compounds **5a** and **5d** exhibited promising cytotoxic activity for both cell lines. For this reason, compounds **5a** and **5d** were pursued for further studies. Starting with determination of IC_50s_ and cytotoxic selectivity studies. Serial concentrations of compounds **5a** and **5d** were used to examine their impact on cell viability using MTT protocol. Results of concentration *vs* percent viability were charted, and the IC_50_ was calculated for SW-620 and HT-29 cell lines using Graph Pad prism 8 (Figure 4). Compound **5a** was found to have IC_50_ = 9.4 µM and 8.7 µM against SW-620 and HT-29 cell lines, respectively. In addition, the IC_50_ for compound **5d** equals 9.8 µM and 6.5 µM against SW-620 and HT-29 cell lines, respectively, compared to IC_50_ of **Irinotecan**, a reference drug, which was found to be 1.0 µM against SW-620 cell line and 6.18 µM against HT-29 cell line (Figure 4**)**.

**Figure 5.**
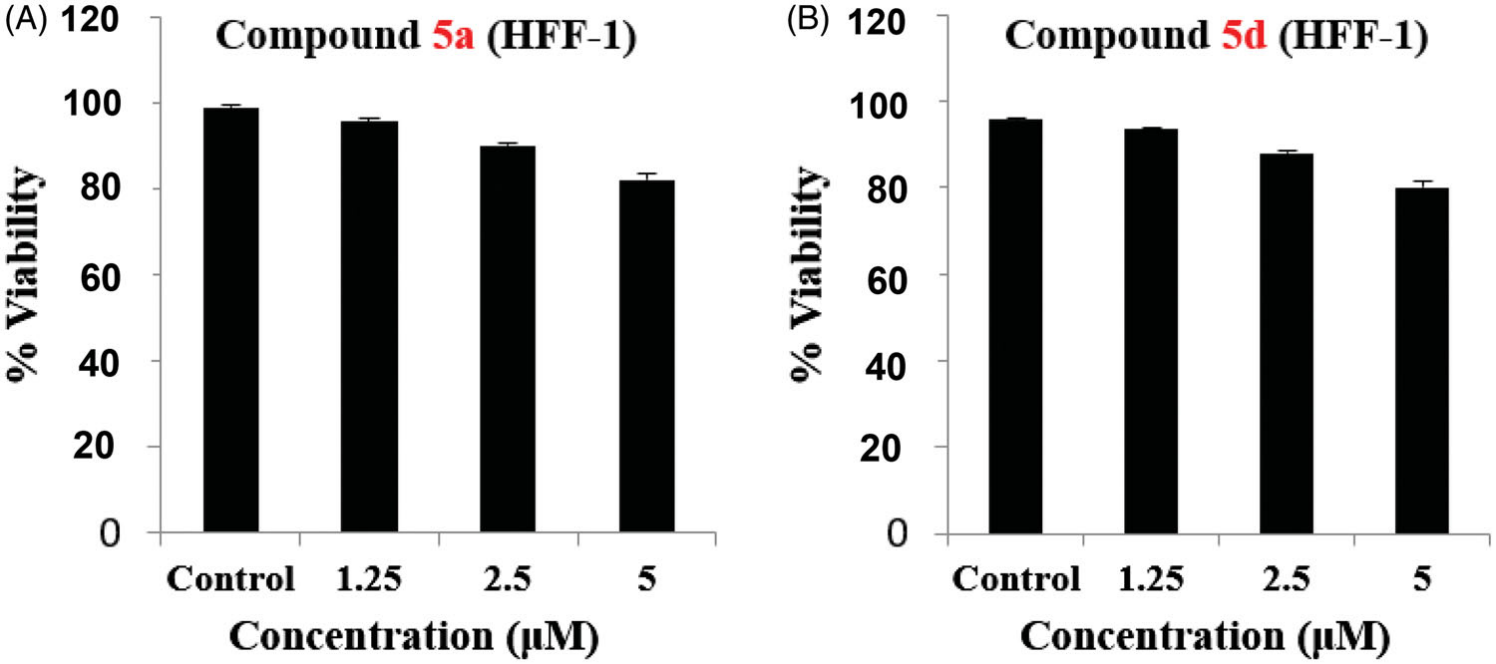
Impact of **5a** and **5d** on normal HFF-1 fibroblast cells, upon incubation for 24 h. (A) compound **5a** and (B) compound **5d**.

Furthermore, selective cytotoxicity of compounds **5a** and **5d** was studied on human skin fibroblast (HFF-1) normal cells. Both conjugates were found to possess a little effect on fibroblast normal cell viability (Figure 5). These results revealed that compounds **5a** and **5d** possessed a selective cytotoxicity against SW-620 and HT-29 cancer cell lines with non-significant effect on normal fibroblast cells.

#### 2.2.2. Annexin V-FITC/propidium iodide apoptosis assay

**Figure 6.**
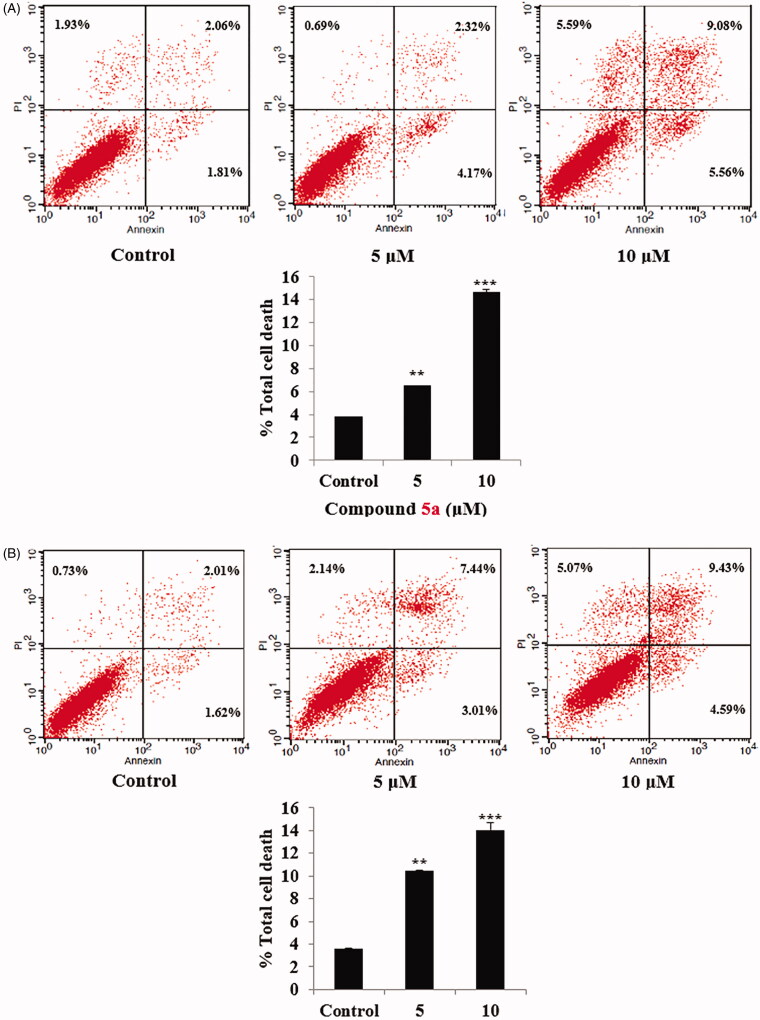
(A) AnnexinV/PI apoptosis assay for compound **5a**. Tow concentrations (5 and 10 µM) of compound **5a**, in addition untreated plate as a control were used to test the apoptotic effect by using Annexin V/PI in SW-620 cell line. Cells were treated with the compound **5a** for 24 h. (B) AnnexinV/PI apoptosis assay for compound 5d. Tow concentrations (5 and 10 µM) of compound **5d**, in addition untreated plate as a control were used to test the apoptotic effect by using Annexin V/PI in SW-620 cell line. Cells were treated with the compound **5d** for 24 h.

Further investigation for compounds **5a** and **5d** concerning their potential role of apoptosis induction, using Annexin V-FITC/PI double staining assay^61^, was performed to evaluate their impact on both early and late apoptosis percentages in SW-620 cancer cell lines (Figure 6). The assay findings showed that compounds **5a** and **5d** resulted in a dose dependent induction of apoptosis for SW-620 cancer cells. As shown, compound **5a** induced approximately 1.7-folds and 3.8-folds total increase in apoptosis at concentration of 5 µM and 10 µM, respectively, in comparison to the control untreated SW-620 cell line (Figure 6(A)).

Similarly, compound **5d**, at concentration of 5 µM and 10 µM approximately induced 2.9-folds and 3.8-folds total increase in apoptosis, respectively, when incubated with SW-620 cell line, compared to the untreated cells (Figure 6(B)). Encouraged by these results compounds **5a** and **5d** were further investigated for their effect on the anti-apoptotic mitochondrial protein Bcl2 and their effect on the level of cleaved PARP in SW-620 colorectal cancer cell line.

#### 2.2.3. Effect of compounds 5a and 5d on the anti-apoptotic markers Bcl2 and the level of cleaved PARP

**Figure 7.**
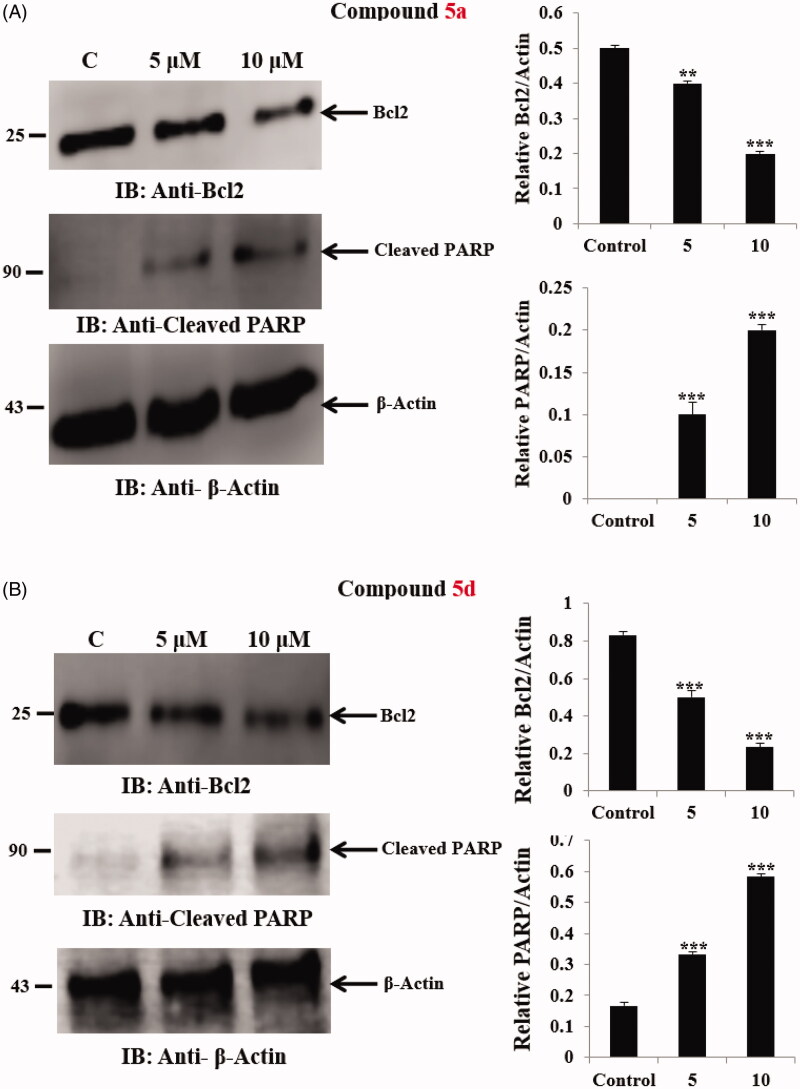
(A) Effect of hybrid 5a on anti-apoptotic Bcl2 protein and the level of cleaved PARP. Statistical analysis was performed where the significance of data was assessed at a *p* values* < 0.05*. *** *p* < 0.001; ** *p* < 0.01 control *vs* treated. (B) Effect of hybrid 5d on anti-apoptotic Bcl2 protein and the level of cleaved PARP. Statistical analysis was performed where the significance of data was assessed at a *p* values* < 0.05*. *** *p* < 0.001; ** *p* < 0.01 control *vs* treated.

To further examine the possible mechanism of apoptosis, the effect of compounds **5a** and **5d** on certain apoptosis-related proteins was studied. Bcl2 protein as a critical component of the mitochondrial apoptotic pathway is reported to be overexpressed in numerous tumours causing survival of cancer cell^62^. In addition, it was reported that caspase activation during apoptosis leads to proteolytic cleavage of several cellular substrates participating in DNA reparation including [poly (ADP-ribose) polymerase]^63^. Therefore, the impact of compounds **5a** and **5d** on the anti-apoptotic protein Bcl2 and the level of cleaved PARP was examined (Figure 7). The results showed that, Western blot analysis of the extracts prepared from SW-620 cells incubated with compound **5a** (5 μM and 10 μM) for 24 h, resulted in a dose dependent inhibition of Bcl2 protein expression and significant increase in the level of cleaved PARP (Figure 7(A)).

Similarly, compound **5d** was found to follow the same pattern with significant inhibition of the anti-apoptotic Bcl2 protein expression and significant increase in the level of cleaved PARP in SW-620 cancer cells (Figure 7(B)). These findings indicated that both compounds **5a** and **5d** inhibited SW-620 cells viability by deregulating apoptosis-related proteins (anti-apoptotic Bcl2 and cleaved PARP) resulting in the induction of apoptosis.

## 3. Conclusions

In summary, a novel series of benzofuran-isatin conjugates linked by a carbohydrazide group, (**5a–e** and **7a–i**) was designed and synthesised. Seven compounds (**5b–d** and **7a,b,d,g**) were selected according to NCI’s DTP selection guidelines for the assessment of their antitumor activity against NCI-55 human cancer cell lines. All compounds proved effective against diverse cell lines among which compound **5d** was promoted to the five-dose screen and showed good to excellent growth inhibitory activity against almost all subpanel cancer cell lines. In addition, the novel conjugates (**5a–e** and **7a–i**) showed good anti-proliferative activity against two human colorectal cancer cell lines, SW-620 and HT-29, with excellent inhibitory activity for compounds **5a** and **5d** that showed IC_50_ = 8.7 µM and 9.4 µM for **5a** and IC_50_ = 6.5 µM and 9.8 µM for **5d** against SW-620 and HT-29 cell lines, respectively, and proved to have selective cytotoxicity with increased safety profile to fibroblast (HFF-1) normal cells. Further mechanistic studies revealed that both compounds **5a** and **5d** were able to induce apoptosis in a dose dependent manner with an approximately 1.7–3.8 folds and 2.9–3.8 folds total increase in apoptosis for compounds **5a** and **5d**, respectively, compared to the control untreated SW-620 cell line. Furthermore, both conjugates significantly inhibited the expression of the anti-apoptotic Bcl2 protein and increased the level of the cleaved PARP and resulted in SW-620 cells apoptosis. Collectively, the significant potency and high selective cytotoxicity of this series specially compounds **5a** and **5d** suggested that these conjugates might serve as starting point for additional optimisation to develop potential anticancer agents and apoptotic inducers.

## 4. Experimental

### 4.1. Chemistry

#### 4.1.1. General

Solvents of HPLC grade have been used and purchased from Thermo Fisher. Follow up of reactions has been performed utilising precoated TLC F_254_ Merck plates. Schimadzu FT-IR spectrometer has been used for functional groups analysis for the synthesised derivatives. NMR spectrometric analyses have been conducted using Bruker-Avance 400 NMR spectrometer (100 MHz for ^13^CNMR and 400 MHz for ^1^H NMR). Chemical shifts have been recorded in *ppm*. Multiplicities have been reported with their 1st order J coupling constants (Hz) for doublets (d); Stuart apparatus has been used to determine the melting points. FLASH 2000 CHNS/O analyser has been adopted to perform the elemental analysis. Compounds **3**^64^, and **6a–i**^65^^,^^66^ have been reported previously.

#### 4.1.2. Synthesis of target derivatives 5a–e and 7a–i

To stirred hot solution of 3-methylbenzofuran-2-carbohydrazide **3** (0.25 g, 1.3 mmol) in 13 ml of absolute EtOH with catalytic drops of ethanoic acid, equivalent amount of appropriate indoline-2,3-dione compounds **4a–e** or **6a–i** has been added. The reaction mixture has been then refluxed for (3–6) h. The produced precipitate, after cooling, was collected by filtration, washed with water then recrystallized from glacial acetic acid to produce target derivatives **5a–e** and **7a–i**, respectively in a good yield (70–87%).

Full characterisation (NMR, IR, and elemental analysis) data for target compounds (**5a–e** and **7a–i**) have been presented in the Supporting Materials.

### 4.2. Biological evaluation

All *in vitro* biological assays in this study; NCI anticancer screening^67^^,^^68^, MTT cell viability assay^54^, Annexin V-FITC/PI assay^54^ and Western blot analysis^54^ were performed as reported earlier. All experimental procedures were provided in the Supporting materials.

## Supplementary Material

Supplemental Material

## References

[1] Siegel RL, Miller KD, Jemal A. Cancer statistics, 2016. CA Cancer J Clin 2016;66:7–30. 26742998 10.3322/caac.21332

[2] Siegel RL, Miller KD, Jemal A. Cancer statistics, 2020. CA Cancer J. Clin 2020;70:7–30. 31912902 10.3322/caac.21590

[3] Cheung-Ong K, Giaever G, Nislow C. DNA-damaging agents in cancer chemotherapy: serendipity and chemical biology. Chem Biol 2013;20:648–59. 23706631 10.1016/j.chembiol.2013.04.007

[4] DeVita VT Jr., Chu E. A history of cancer chemotherapy. Cancer Res 2008;68:8643–53. 18974103 10.1158/0008-5472.CAN-07-6611

[5] Fischhaber PL, Gall AS, Duncan JA, Hopkins PB. Direct demonstration in synthetic oligonucleotides that N,N′-bis(2-chloroethyl)-nitrosourea cross links N1 of deoxyguanosine to N3 of deoxycytidine on opposite strands of duplex DNA. Cancer Res 1999;59:4363–8. 10485484

[6] Goodman LS, Wintrobe MM. Nitrogen mustard therapy; use of methyl-bis (beta-chloroethyl) amine hydrochloride and tris (beta-chloroethyl) amine hydrochloride for Hodgkin’s disease, lymphosarcoma, leukemia and certain allied and miscellaneous disorders. J Am Med Assoc 1946;132:126–32. 20997191 10.1001/jama.1946.02870380008004

[7] Farber S, Diamond LK. Temporary remissions in acute leukemia in children produced by folic acid antagonist, 4-aminopteroyl-glutamic acid. N Engl J Med 1948;238:787–93. 18860765 10.1056/NEJM194806032382301

[8] Nitiss JL. DNA topoisomerases in cancer chemotherapy: using enzymes to generate selective DNA damage. Curr Opin Investig Drugs 2002;3:1512–6. 12431029

[9] Nitiss JL. Targeting DNA topoisomerase II in cancer chemotherapy. Nat Rev Cancer 2009;9:338–50. 19377506 10.1038/nrc2607 PMC2748742

[10] Wadler S, Fuks JZ, Wiernik PH. Phase I and II agents in cancer therapy: I. Anthracyclines and related compounds. J Clin Pharmacol 1986;26:491–509. 2944917 10.1002/j.1552-4604.1986.tb02942.x

[11] Espinosa E, Zamora P, Feliu J, González Barón M. Classification of anticancer drugs–a new system based on therapeutic targets. Cancer Treat. Rev 2003;29:515–23. 14585261 10.1016/s0305-7372(03)00116-6

[12] Mansoori B, Mohammadi A, Davudian S, et al. The different mechanisms of cancer drug resistance: a brief review. Adv Pharm Bull 2017;7:339–48. 29071215 10.15171/apb.2017.041 PMC5651054

[13] Baudino TA. Targeted cancer therapy: the next generation of cancer treatment. Curr Drug Discov Technol 2015;12:3–20. 26033233 10.2174/1570163812666150602144310

[14] Topcul M, Cetin I. Endpoint of cancer treatment: targeted therapies, Asian Pac. Asian Pac J Cancer Prev 2014;15:4395–403. 24969859 10.7314/apjcp.2014.15.11.4395

[15] Modugno M, Banfi P, Gasparri F, et al. Mcl-1 antagonism is a potential therapeutic strategy in a subset of solid cancers. Exp Cell Res 2015;332:267–77. 25486070 10.1016/j.yexcr.2014.11.022

[16] Placzek WJ, Wei J, Kitada S, et al. A survey of the anti-apoptotic Bcl-2 subfamily expression in cancer types provides a platform to predict the efficacy of Bcl-2 antagonists in cancer therapy. Cell Death Dis 2010;1:e40. 21364647 10.1038/cddis.2010.18 PMC3032312

[17] Bai P. Biology of Poly(ADP-Ribose) polymerases: the factotums of cell maintenance. Mol Cell 2015;58:947–58. 26091343 10.1016/j.molcel.2015.01.034

[18] Herceg Z, Wang ZQ. Functions of poly(ADP-ribose) polymerase (PARP) in DNA repair, genomic integrity and cell death. Mutat Res 2001;477:97–110. 11376691 10.1016/s0027-5107(01)00111-7

[19] Langelier MF, Pascal JM. PARP-1 mechanism for coupling DNA damage detection to poly(ADP-ribose) synthesis. Curr Opin Struct Biol 2013;23:134–43. 23333033 10.1016/j.sbi.2013.01.003 PMC3572337

[20] Yu SW, Andrabi SA, Wang H, et al. Apoptosis-inducing factor mediates poly(ADP-ribose) (PAR) polymer-induced cell death. Proc Natl Acad Sci USA 2006;103:18314–9., 17116881 10.1073/pnas.0606528103 PMC1838748

[21] DeSimone RW, Currie KS, Mitchell SA, et al. Privileged structures: applications in drug discovery. Comb Chem High Throughput Screen 2004;7:473–94. 15320713 10.2174/1386207043328544

[22] Khanam H. Shamsuzzaman, Bioactive Benzofuran derivatives: a review. Eur. J. Med. Chem 2015;97:483–504. 25482554 10.1016/j.ejmech.2014.11.039

[23] Nevagi RJ, Dighe SN, Dighe SN. Biological and medicinal significance of benzofuran. Eur J Med Chem 2015;97:561–81. 26015069 10.1016/j.ejmech.2014.10.085

[24] Dawood KM. An update on benzofuran inhibitors: a patent review. Expert Opin Ther Pat 2019;29:841–70. 31560232 10.1080/13543776.2019.1673727

[25] Miao Y-h, Hu Y-h, Yang J, et al. Natural source, bioactivity and synthesis of benzofuran derivatives. RSC Adv 2019;9:27510–40. 10.1039/c9ra04917g PMC9070854 35529241

[26] Radadiya A, Shah A. Bioactive benzofuran derivatives: an insight on lead developments, radioligands and advances of the last decade. Eur J Med Chem 2015;97:356–76. 25703339 10.1016/j.ejmech.2015.01.021

[27] Goyal D, Kaur A, Goyal B. Benzofuran and Indole: promising scaffolds for drug development in Alzheimer’s Disease. ChemMedChem 2018;13:1275–99. 29742314 10.1002/cmdc.201800156

[28] Hiremathad A, Patil MR, K. R C, et al. Benzofuran: an emerging scaffold for antimicrobial agents. RSC Adv 2015;5:96809–28.

[29] Xu Z, Zhao S, Lv Z, et al. Benzofuran derivatives and their anti-tubercular, anti-bacterial activities. Eur J Med Chem 2019;162:266–76. 30448416 10.1016/j.ejmech.2018.11.025

[30] Chand, Rajeshwari K, Hiremathad A, Singh M, et al. A review on antioxidant potential of bioactive heterocycle benzofuran: natural and synthetic derivatives. Pharmacol. Rep 2017;69:281–95. 28171830 10.1016/j.pharep.2016.11.007

[31] Alizadeh M, Jalal M, Hamed K, et al. Recent updates on anti-inflammatory and antimicrobial effects of furan natural derivatives. J Inflamm Res 2020;13:451–63. 32884326 10.2147/JIR.S262132 PMC7443407

[32] Kwiecień H, Goszczyńska A, Rokosz P. Benzofuran small molecules as potential inhibitors of human protein kinases: a review. Curr Pharm Des 2016;22:879–94. 26648467 10.2174/1381612822666151209152457

[33] Flynn BL, Gill GS, Grobelny DW, et al. Discovery of 7-hydroxy-6-methoxy-2-methyl-3-(3,4,5-trimethoxybenzoyl)benzo[b]furan (BNC105), a tubulin polymerization inhibitor with potent antiproliferative and tumor vascular disrupting properties. J Med Chem 2011;54:6014–27. 21774499 10.1021/jm200454y PMC3172808

[34] Romagnoli R, Baraldi PG, Carrion MD, et al. Design, synthesis and structure-activity relationship of 2-(3′,4′,5′-trimethoxybenzoyl)-benzo[b]furan derivatives as a novel class of inhibitors of tubulin polymerization. Bioorg Med Chem 2009;17:6862–71. 19736015 10.1016/j.bmc.2009.08.027 PMC2762272

[35] Xia Y, Jin Y, Kaur N, et al. HIF-1α inhibitors: synthesis and biological evaluation of novel moracin O and P analogues. Eur J Med Chem 2011;46:2386–96. 21481991 10.1016/j.ejmech.2011.03.022

[36] Xie F, Zhu H, Zhang H, et al. In vitro and in vivo characterization of a benzofuran derivative, a potential anticancer agent, as a novel Aurora B kinase inhibitor. Eur J Med Chem 2015;89:310–9. 25462247 10.1016/j.ejmech.2014.10.044

[37] Abdelhafez OM, Amin KM, Ali HI, et al. Design, synthesis and anticancer activity of benzofuran derivatives targeting VEGFR-2tyrosine kinase. RSC Adv 2014;4:11569–79.

[38] Choi MJ, Jung KH, Kim D, et al. Anti-cancer effects of a novel compound HS-113 on cell growth, apoptosis, and angiogenesis in human hepatocellular carcinoma cells. Cancer Lett 2011;306:190–6. 21463918 10.1016/j.canlet.2011.03.005

[39] Gao C, Sun X, Wu Z, et al. A novel Benzofuran derivative Moracin N induces autophagy and apoptosis through ROS Generation In Lung Cancer. Front Pharmacol 2020;11:391. 32477104 10.3389/fphar.2020.00391 PMC7235196

[40] Manna SK, Bose JS, Gangan V, et al. Novel derivative of benzofuran induces cell death mostly by G2/M cell cycle arrest through p53-dependent pathway but partially by inhibition of NF-kappaB. J Biol Chem 2010;285:22318–27. 20472557 10.1074/jbc.M110.131797 PMC2903425

[41] Abd El-Karim SS, Anwar MM, Mohamed NA, et al. Design, synthesis, biological evaluation and molecular docking studies of novel benzofuran-pyrazole derivatives as anticancer agents. Bioorg Chem 2015;63:1–12. 26368040 10.1016/j.bioorg.2015.08.006

[42] Siddiqui SK, SahayaSheela VJ, Kolluru S, et al. Discovery of 3-(benzofuran-2-ylmethyl)-1H-indole derivatives as potential autophagy inducers in cervical cancer cells. Bioorg Med Chem Lett 2020;30:127431. 32769048 10.1016/j.bmcl.2020.127431

[43] Mao ZW, Zheng X, Lin YP, et al. Design, synthesis and anticancer activity of novel hybrid compounds between benzofuran and N-aryl piperazine. Bioorg Med Chem Lett 2016;26:3421–4. 27371110 10.1016/j.bmcl.2016.06.055

[44] Xu K, Liu Y, Wang R, et al. Design, synthesis, and anticancer activities of Benzofuran–isatin hybrids tethered by pentylene and hexylene. J. Hetero. Chem 2019;56:2052–5.

[45] De Moraes G, Teixeira PA, Pena LJ, Leite ACL. Isatin derivatives and their antiviral properties against arboviruses: a review. Mini Rev Med Chem 2019;19:56–62. 29692243 10.2174/1389557518666180424093305

[46] Guo H. Isatin derivatives and their anti-bacterial activities. Eur J Med Chem 2019;164:678–88. 30654239 10.1016/j.ejmech.2018.12.017

[47] Mathur G, Nain S. Recent advancement in synthesis of isatin as anticonvulsant agents, a review. Med Chem 2014;4:417–27.

[48] Phogat P, Singh P. A mini review on central nervous system potential of isatin derivatives. Cent Nerv Syst Agents Med Chem 2015;15:28–31. 25693647 10.2174/1871524915666150213122246

[49] Ding Z, Zhou M, Zeng C. Recent advances in isatin hybrids as potential anticancer agents. Arch Pharm 2020;353:e1900367 10.1002/ardp.201900367 31960987

[50] Hou Y, Shang C, Wang H, Yun J. Isatin-azole hybrids and their anticancer activities. Arch Pharm 2020;353:e1900272. 10.1002/ardp.201900272 31691360

[51] Abdel-Aziz HA, Eldehna WM, Keeton AB, et al. Isatin-benzoazine molecular hybrids as potential antiproliferative agents: synthesis and *in vitro* pharmacological profiling. Drug Des Devel Ther 2017;11:2333–46. 10.2147/DDDT.S140164 PMC5557401 28848327

[52] Eldehna WM, El Hassab MA, Abo-Ashour MF, et al. Development of isatin-thiazolo[3,2-a]benzimidazole hybrids as novel CDK2 inhibitors with potent in vitro apoptotic anti-proliferative activity: synthesis, biological and molecular dynamics investigations. Bioorg Chem 2021;110:104748. 33684714 10.1016/j.bioorg.2021.104748

[53] El-Naggar M, Eldehna WM, Almahli H, et al. Novel Thiazolidinone/Thiazolo[3,2-a]Benzimidazolone-Isatin conjugates as apoptotic anti-proliferative agents towards breast cancer: one-pot synthesis and *in vitro* biological evaluation. Molecules 2018;23:1420. 10.3390/molecules23061420 PMC6099623 29895744

[54] Eldehna WM, Abo-Ashour MF, Al-Warhi T, et al. Development of 2-oindolin-3-ylidene-indole-3-carbohydrazide derivatives as novel apoptotic and anti-proliferative agents towards colorectal cancer cells. J Enzy Inhib Med Chem 2021;36:319–28. 10.1080/14756366.2020.1862100 PMC7751403 33345633

[55] Fares M, Eldehna WM, Abou-Seri SM, et al. Design, synthesis and *in vitro* antiproliferative activity of novel isatin-quinazoline hybrids. Arch Pharm 2015;348:144–54. 10.1002/ardp.201400337 25664631

[56] Montreal Q. Operating Environment (MOE), 10. Montreal: Chemical Computing Group Inc; 2009.

[57] Boyd MR, Paull KD. Some practical considerations and applications of the national cancer institute in vitro anticancer drug discovery screen. Drug Dev. Res 1995;34:91–109.

[58] Shoemaker RH. The NCI60 human tumour cell line anticancer drug screen. Nat Rev Cancer 2006;6:813–23. 16990858 10.1038/nrc1951

[59] Acton EM, Narayanan VL, Risbood PA, et al. Anticancer specificity of some ellipticinium salts against human brain tumors in vitro. J Med Chem 1994;37:2185–9. 8035425 10.1021/jm00040a010

[60] Mosmann T. Rapid colorimetric assay for cellular growth and survival: application to proliferation and cytotoxicity assays. J Immunol Methods 1983;65:55–63. 6606682 10.1016/0022-1759(83)90303-4

[61] Rieger AM, Nelson KL, Konowalchuk JD, Barreda DR. Modified annexin V/propidium iodide apoptosis assay for accurate assessment of cell death. J. Vis. Exp 2011;(50):2597–603. 10.3791/2597 PMC3169266 21540825

[62] Marone M, Ferrandina G, Macchia G, et al. Bcl-2, Bax, Bcl-x(L) and Bcl-x(S) expression in neoplastic and normal endometrium. Oncology 2000;58:161–8. 10705244 10.1159/000012094

[63] Fischer U, Janicke RU, Schulze-Osthoff K. Many cuts to ruin: a comprehensive update of caspase substrates. Cell Death Differ 2003;10:76–100. 12655297 10.1038/sj.cdd.4401160 PMC7091709

[64] Eldehna WM, Nocentini A, Elsayed ZM, et al. Benzofuran-based carboxylic acids as carbonic anhydrase inhibitors and antiproliferative agents against breast cancer. ACS Med Chem Lett 2020;11:1022–7. 32435420 10.1021/acsmedchemlett.0c00094 PMC7236537

[65] Al-Warhi T, El Kerdawy AM, Aljaeed N, et al. Synthesis, biological evaluation and in silico studies of certain oxindole-indole conjugates as anticancer CDK inhibitors. Molecules 2020;25:2031–9. 10.3390/molecules25092031 PMC7248897 32349307

[66] Elsayed ZM, Eldehna WM, Abdel-Aziz MM, et al. Development of novel isatin-nicotinohydrazide hybrids with potent activity against susceptible/resistant Mycobacterium tuberculosis and bronchitis causing-bacteria. J Enzyme Inhib Med Chem 2021;36:384–93. 33406941 10.1080/14756366.2020.1868450 PMC7801109

[67] Al-Rashood ST, Hamed AR, Hassan GS, et al. Antitumor properties of certain spirooxindoles towards hepatocellular carcinoma endowed with antioxidant activity. J Enzyme Inhib Med Chem 2020;35:831–9. 32208781 10.1080/14756366.2020.1743281 PMC7144320

[68] Al-Warhi T, Abo-Ashour MF, Almahli H, et al. Novel [(N-alkyl-3-indolylmethylene)hydrazono]oxindoles arrest cell cycle and induce cell apoptosis by inhibiting CDK2 and Bcl-2: synthesis, biological evaluation and in silico studies. J Enzyme Inhib Med Chem 2020;35:1300–9. 32522063 10.1080/14756366.2020.1773814 PMC7717600

