# Supplementary material for: Development of novel benzofuran-isatin conjugates as potential antiproliferative agents with apoptosis inducing mechanism in Colon cancer
Source: J Enzyme Inhib Med Chem. 2021 Jun 28;36(1):1424–35. doi: 10.1080/14756366.2021.1944127 (PMC8245078; doi:10.1080/14756366.2021.1944127)
Supplement: Supplemental Material [file IENZ_A_1944127_SM4352.pdf]

Supporting Information

Development of Novel Benzofuran-Isatin Conjugates as Potential Antiproliferative Agents with Apoptosis Inducing Mechanism in Colon Cancer

Wagdy M. Eldehna*, Rofaida Salem, Zainab M. Elsayed, Tarfah Al-Warhi, Hamada R. Knany, Rezk R. Ayyad[e], Thamer Bin Traiki, Maha Abdulla, Rehan Ahmad, Hatem A. Abdel-Aziz*, Radwan El-Haggar

Tables of Contents

**1.**	**Physical and spectral data for target compounds *5a-e* and *7a-i***	3
**2.**	**Biological Evaluation**	8
	2.1. NCI, USA Anticancer Screening	8
	2.2. Cell Culture	9
	2.3. Cell Viability Assay	10
	2.4. Measurement of Apoptosis by Annexin V-FITC/PI Assay	10
	2.5. Western Blot Analysis	10
	2.6. Statistical Analysis	11
**3.**	**NMR Spectra**	12-30

1. Physical and spectral data for target compounds *5a-e* and *7a-i*

1.1. 3-Methyl-N'-(2-oxoindolin-3-ylidene)benzofuran-2-carbohydrazide ***5a***.

Yield 80%, m.p. > 300 °C; ^{1}H NMR *δ ppm*: 2.64 (s, 3H, CH_3), 6.96 (t, 1H, Ar-H, *J*= 8.4 Hz), 7.12-7.22 (m, 1H, Ar-H), 7.40-7.49 (m, 2H, Ar-H), 7.57-7.64 (m, 1.7H, Ar-H), 7.69 (d, 0.3H, Ar-H, *J*= 8.4 Hz), 7.78 (d, 0.7H, Ar-H, *J*= 8.4 Hz), 7.84 (d, 1H, Ar-H, *J*= 7.6 Hz), 7.93 (d, 0.3H, Ar-H, *J*= 7.6 Hz), 10.91, 11.60 (2s, 1H, NH isatin, D_2O exchangeable), 11.37, 14.05 (2s, 1H, NH, D_2O exchangeable); ^{13}C NMR *δ ppm*: 9.43, 9.46 (CH_3), 111.41, 111.72, 112.32, 112.59, 116.21, 120.31, 121.55, 121.98, 122.07, 122.56, 123.22, 124.13, 124.25, 125.02, 125.80, 126.77, 128.64, 129.00, 129.26, 132.42, 133.59, 138.85, 141.67, 142.16, 142.70, 143.13, 144.53, 153.49, 153.66, 156.30 (Aromatic carbons), 163.30 (C=O of hydrazide), 165.05 (C=O of isatin); IR (KBr, *ν* cm^{-1}) 3301, 3250 (2NH) and 1711, 1693 (2C=O); MS *m/z* [%]: 319 [M^{+}, 83]; Anal. Calcd. for $C_{18}H_{13}N_3O_3$ (319.3): C, 67.71; H, 4.10; N, 13.16; found C, 67.77; H, 4.14; N, 13.11.

1.2. N'-(5-Fluoro-2-oxoindolin-3-ylidene)-3-methylbenzofuran-2-carbohydrazide ***5b***.

Yield 72%, m.p. > 300 °C; ^{1}H NMR *δ ppm*: 2.63 (s, 3H, CH_3), 6.93-6.97 (m, 1H, Ar-H), 7.31 (t, 1H, Ar-H, *J*= 8.0 Hz), 7.40 (t, 1H, Ar-H, *J*= 8.0 Hz),7.56 (t, 1H, Ar-H, *J*= 8.0 Hz), 7.72-7.78 (m, 2H, Ar-H), 7.84 (d, 1H, Ar-H, *J*= 8.0 Hz), 10.93 (s, 1H, NH isatin, D_2O exchangeable), 11.76 (s, 1H, NH, D_2O exchangeable); ^{13}C NMR *δ ppm*: 9.49 (CH_3), 112.22, 112.44, 113.89, 116.41, 119.70, 121.99, 124.12, 125.12, 128.72, 129.21, 140.87, 142.20, 153.68, 156.55, 158.90 (Aromatic carbons), 163.39 (C=O of hydrazide), 165.11 (C=O of isatin); IR (KBr, *ν* cm^{-1}) 3317, 3242 (2NH) and 1721, 1704 (2C=O); MS *m/z* [%]: 337 [M^{+}, 53]; Anal. Calcd. for $C_{18}H_{12}FN_3O_3$ (337.3): C, 64.09; H, 3.59; N, 12.46; found C, 64.01; H, 3.52; N, 12.41.

1.3. N'-(5-Bromo-2-oxoindolin-3-ylidene)-3-methylbenzofuran-2-carbohydrazide ***5c***.

Yield 84%, m.p. > 300 °C; ^{1}H NMR *δ ppm*: 2.67 (s, 3H, CH_3), 7.24 (d, 1H, Ar-H, *J*= 8.0 Hz), 7.44 (t, 1H, Ar-H, *J*= 8.0 Hz), 7.60 (t, 1H, Ar-H, *J*= 8.0 Hz), 7.70 (d, 1H, Ar-H, *J*= 8.0 Hz), 7.75-7.80 (m, 2H, Ar-H), 7.87 (d, 1H, Ar-H, *J*= 8.0 Hz), 10.97 (s, 1H, NH isatin, D_2O exchangeable), 13.98 (s, 1H, NH, D_2O exchangeable); ^{13}C NMR *δ ppm*: 9.46 (CH_3), 112.15, 113.23, 113.69, 117.80, 122.10, 124.16, 125.04, 128.79, 129.27, 135.59, 141.49, 142.41, 143.57, 153.61, 158.24 (Aromatic carbons), 162.16 (C=O of hydrazide), 164.67 (C=O of isatin); IR

(KBr, *ν* cm^{-1}) 3280, 3220 (2NH) and 1737, 1724 (2C=O); Anal. Calcd. for $C_{18}H_{12}BrN_3O_3$ (398.2): C, 54.29; H, 3.04; N, 10.55; found C, 54.21; H, 3.00; N, 10.51.

1.4. N'-(5-Methoxy-2-oxoindolin-3-ylidene)-3-methylbenzofuran-2-carbohydrazide ***5d***.

Yield 82%, m.p. 238-239 °C; ^{1}H NMR *δ ppm*: 2.66 (s, 3H, CH3), 3.80 (s, 3H, OCH3), 6.89 (d, 1H, Ar-H, *J*= 8.0 Hz), 6.99 (br s, 1H, Ar-H), 7.15 (s, 1H, Ar-H), 7.42 (s, 1H, Ar-H), 7.59 (br s, 1H, Ar-H), 7.69 (d, 1H, Ar-H, *J*= 8.0 Hz), 7.84 (d, 1H, Ar-H, *J*= 8.0 Hz), 11.18 (s, 1H, NH isatin, D_2O exchangeable), 14.10 (s, 1H, NH, D_2O exchangeable); ^{13}C NMR *δ ppm*: 9.43 (CH_3), 56.11 (OCH_3), 112.33, 112.63, 118.79, 121.06, 122.08, 124.24, 129.03, 130.94, 136.80, 139.14, 153.32, 156.31, 158.63 (Aromatic carbons), 163.40 (C=O of hydrazide), 166.44 (C=O of isatin); IR (KBr, *ν* cm^{-1}) 3248, 3210 (2NH) and 1730, 1722 (2C=O); MS *m/z* [%]: 349 [M$^+$, 71]; Anal. Calcd. for $C_{19}H_{15}N_3O_4$ (349.3): C, 65.32; H, 4.33; N, 12.03; found C, 65.25; H, 4.30; N, 12.09.

1.5. N'-(5-Nitro-2-oxoindolin-3-ylidene)-3-methylbenzofuran-2-carbohydrazide ***5e***.

Yield 89%, m.p. > 300 °C; ^{1}H NMR *δ ppm*: 2.65 (s, 3H, CH_3), 7.12-7.15 (m, 1H, Ar-H), 7.39-7.45 (m, 1H, Ar-H), 7.57-7.70 (m, 2H, Ar-H), 7.82-7.89 (m, 1H, Ar-H), 8.24 (s, 0.3H, Ar-H), 8.28 (d, 0.3H, Ar-H, *J*= 8.0 Hz), 8.37 (d, 0.7H, Ar-H, *J*= 8.0 Hz), 8.90 (s, 0.7H, Ar-H), 11.61, 11.98 (2s, 1H, NH isatin, D_2O exchangeable), 12.09, 13.82 (2s, 1H, NH, D_2O exchangeable); ^{13}C NMR *δ ppm*: 9.47 (CH_3), 111.46, 112.12, 115.90, 122.49, 124.23, 126.50, 128.98, 137.10, 140.60, 141.35, 142.25, 143.26, 148.16, 149.88, 153.67 (Aromatic carbons), 163.62 (C=O of hydrazide), 165.32 (C=O of isatin); IR (KBr, *ν* cm^{-1}) 3262, 3205 (2NH) and 1716, 1698 (2C=O); MS *m/z* [%]: 364 [M$^+$, 39]; Anal. Calcd. for $C_{18}H_{12}N_4O_5$ (364.3): C, 59.34; H, 3.32; N, 15.38; found C, 59.39; H, 3.37; N, 15.32.

1.6. 3-Methyl-N'-(1-methyl-2-oxoindolin-3-ylidene)benzofuran-2-carbohydrazide ***7a***.

Yield 83%, m.p. 262-264 °C; ^{1}H NMR *δ ppm*: 2.66 (s, 3H, CH_3), 3.28 (s, 3H, *N*-CH_3), 7.40 (t, 1H, Ar-H, *J*= 8.0 Hz), 7.49 (t, 1H, Ar-H, *J*= 8.0 Hz), 7.57 (t, 1H, Ar-H, *J*= 8.0 Hz), 7.65 (d, 1H, Ar-H, *J*= 8.0 Hz), 7.71 (d, 1H, Ar-H, *J*= 8.0 Hz), 7.84 (d, 1H, Ar-H, *J*= 8.0 Hz), 14.03 (s, 1H, NH, D_2O exchangeable); ^{13}C NMR *δ ppm*: 9.43 (CH_3), 26.27 (*N*-CH_3), 110.54, 112.35, 119.61, 121.21, 122.10, 123.77, 124.29, 125.93, 129.08, 132.35, 141.63, 144.43, 147.01, 153.53, 156.38 (Aromatic carbons), 161.56 (C=O of hydrazide), 166.39 (C=O of isatin); IR (KBr, *ν*

cm^{-1}) 3345 (NH) and 1720, 1695 (2C=O); MS *m/z* [%]: 333 [M$^+$, 92]; Anal. Calcd. for $C_{19}H_{15}N_3O_3$ (333.3): C, 68.46; H, 4.54; N, 12.61; found C, 68.40; H, 4.50; N, 12.64.

*1.7. 3-Methyl-N'-(2-oxo-1-propylindolin-3-ylidene)benzofuran-2-carbohydrazide **7b**.*

Yield 76%, m.p. 181-183 °C; ^{1}H NMR *δ ppm*: 0.93 (t, 3H, -CH$_2$C$\underline{H_3}$, *J*= 8.0 Hz), 1.66-1.75 (m, 2H, -C$\underline{H_2}$CH$_3$), 2.66 (s, 3H, CH$_3$), 3.76 (t, 2H, *N*-CH$_2$, *J*= 8.0 Hz), 7.17 (t, 1H, Ar-H, *J*= 8.0 Hz), 7.25 (d, 1H, Ar-H, *J*= 8.0 Hz), 7.40 (t, 1H, Ar-H, *J*= 8.0 Hz), 7.47 (t, 1H, Ar-H, *J*= 8.0 Hz), 7.57 (t, 1H, Ar-H, *J*= 8.0 Hz), 7.66 (d, 1H, Ar-H, *J*= 8.0 Hz), 7.72 (d, 1H, Ar-H, *J*= 8.0 Hz), 7.84 (d, 1H, Ar-H, *J*= 8.0 Hz), 14.08 (s, 1H, NH, D$_2$O exchangeable); ^{13}C NMR *δ ppm*: 9.42 (CH$_3$), 11.68 (-CH$_2$-$\underline{C}$H$_3$), 20.92 (-$\underline{C}$H$_2$-CH$_3$), 41.30 (*N*-CH$_2$), 110.71, 112.37, 119.66, 123.65, 124.26, 125.96, 129.04, 132.32, 137.97, 141.60, 143.70, 153.52, 156.25, 158.62, 159.00 (Aromatic carbons), 161.50 (C=O of hydrazide), 164.08 (C=O of isatin); IR (KBr, *ν* cm^{-1}) 3304 (NH) and 1715, 1701 (2C=O); Anal. Calcd. for $C_{21}H_{19}N_3O_3$ (361.4): C, 69.79; H, 5.30; N, 11.63; found C, 69.70; H, 5.25; N, 11.67.

*1.8. N'-(1-Allyl-2-oxoindolin-3-ylidene)-3-methylbenzofuran-2-carbohydrazide **7c**.*

Yield 81%, m.p. 214-216 °C; ^{1}H NMR *δ ppm*: 2.52 (s, 3H, CH$_3$), 4.38 (s, 2H, *N*-CH$_2$), 5.15 (d, 1H, CH=C$\underline{H_a}$, *J*= 8.8 Hz), 5.20 (d, 1H, CH=C$\underline{H_b}$, *J*= 15.6 Hz), 5.81-5.91 (m, 1H, *N*-CH$_2$-C$\underline{H}$), 7.02 (d, 1H, Ar-H, *J*= 8.0 Hz), 7.09 (t, 1H, Ar-H, *J*= 8.0 Hz), 7.30-7.38 (m, 2H, Ar-H), 7.47 (t, 1H, Ar-H, *J*= 8.0 Hz), 7.62 (d, 2H, Ar-H, *J*= 8.0 Hz), 7.71 (d, 1H, Ar-H, *J*= 8.0 Hz), 13.96 (s, 1H, NH, D$_2$O exchangeable); ^{13}C NMR *δ ppm*: 8.96 (CH$_3$), 41.76 (*N*-CH$_2$), 110.96, 113.82, 116.67, 117.65, 119.52, 121.18, 121.67, 123.97, 125.85, 129.22, 131.93, 141.56, 143.36, 153.56, 156.18, 158.46, 159.24 (Aromatic carbons), 161.15 (C=O of hydrazide), 164.32 (C=O of isatin); IR (KBr, *ν* cm^{-1}) 3312 (NH) and 1720, 1711 (2C=O); MS *m/z* [%]: 359 [M$^+$, 68]; Anal. Calcd. for $C_{21}H_{17}N_3O_3$ (359.3): C, 70.18; H, 4.77; N, 11.69; found C, 70.10; H, 4.73; N, 11.64.

*1.9. N'-(1-Benzyl-2-oxoindolin-3-ylidene)-3-methylbenzofuran-2-carbohydrazide **7d**.*

Yield 84%, m.p. 228-229 °C; ^{1}H NMR *δ ppm*: 2.68 (s, 3H, CH$_3$), 5.07 (s, 2H, *N*-CH$_2$), 7.08 (d, 1H, Ar-H, *J*= 8.0 Hz), 7.17 (t, 1H, Ar-H, *J*= 8.0 Hz), 7.29 (t, 1H, Ar-H, *J*= 8.0 Hz), 7.36-7.40 (m, 2H, Ar-H), 7.41-7.46 (m, 4H, Ar-H), 7.57 (t, 1H, Ar-H, *J*= 8.0 Hz), 7.69 (d, 1H, Ar-H, *J*= 8.0 Hz), 7.73 (d, 1H, Ar-H, *J*= 8.0 Hz), 7.86 (d, 1H, Ar-H, *J*= 8.0 Hz), 14.01 (s, 1H, NH, D$_2$O exchangeable); ^{13}C NMR *δ ppm*: 9.46 (CH$_3$), 43.07 (*N*-CH$_2$), 111.03, 112.39, 119.84, 121.41,

122.11, 123.91, 124.30, 126.06, 127.87, 128.13, 129.10, 129.23, 129.26, 132.24, 136.13, 137.87, 141.61, 143.36, 153.54, 156.27 (Aromatic carbons), 161.59 (C=O of hydrazide), 165.16 (C=O of isatin); IR (KBr, *ν* cm^{-1}) 3324 (NH) and 1745, 1715 (2C=O); MS *m*/*z* [%]: 409 [M^+, 87]; Anal. Calcd. for $C_{25}H_{19}N_3O_3$ (409.4): C, 73.34; H, 4.68; N, 10.26; found C, 73.39; H, 4.60; N, 10.22.

1.10. N'-(1-(4-Fluorobenzyl)-2-oxoindolin-3-ylidene)-3-methylbenzofuran-2-carbohydrazide ***7e***.

Yield 81%, m.p. 236-238 °C;^{1}H NMR *δ ppm*: 2.60 (s, 3H, CH_3), 4.98 (s, 2H, *N*-CH_2), 7.00 (d, 1H, Ar-H, *J*= 8.0 Hz), 7.07-7.12 (m, 3H, Ar-H), 7.32-7.36 (m, 2H, Ar-H), 7.41-7.44 (m, 2H, Ar-H), 7.48 (t, 1H, Ar-H, *J*= 8.0 Hz), 7.62 (t, 2H, Ar-H, *J*= 8.0 Hz), 7.74 (d, 1H, Ar-H, *J*= 8.0 Hz), 13.93 (s, 1H, NH, D_2O exchangeable); ^{13}C NMR *δ ppm*: 8.81 (CH_3), 42.14 (*N*-CH_2), 110.91, 112.08, 113.76, 116.61, 119.46, 121.20, 123.61, 125.88, 126.94, 127.01, 128.33, 128.60, 129.02, 131.85, 134.95, 143.84, 149.67, 153.58, 156.21, 158.43, 159.21 (Aromatic carbons), 161.53 (C=O of hydrazide), 165.67 (C=O of isatin); IR (KBr, *ν* cm^{-1}) 3309 (NH) and 1712, 1701 (2C=O); MS *m*/*z* [%]: 427 [M^+, 63]; Anal. Calcd. for $C_{25}H_{18}FN_3O_3$ (427.4): C, 70.25; H, 4.24; N, 9.83; found C, 70.32; H, 4.29; N, 9.80.

1.11. N'-(1-(4-Cyanobenzyl)-2-oxoindolin-3-ylidene)-3-methylbenzofuran-2-carbohydrazide ***7f***.

Yield 75%, m.p. 275-277 °C; ^{1}H NMR *δ ppm*: 2.69 (s, 3H, CH_3), 5.13 (s, 2H, *N*-CH_2), 7.04 (d, 1H, Ar-H, *J*= 8.0 Hz), 7.19 (t, 1H, Ar-H, *J*= 8.0 Hz), 7.41 (t, 2H, Ar-H, *J*= 8.0 Hz), 7.58 (t, 2H, Ar-H, *J*= 8.0 Hz), 7.71-7.74 (m, 2H, Ar-H), 7.79 (d, 2H, Ar-H, *J*= 8.0 Hz), 7.86 (d, 1H, Ar-H, *J*= 8.0 Hz), 7.97 (s, 1H, Ar-H), 13.98 (s, 1H, NH, D_2O exchangeable); ^{13}C NMR *δ ppm*: 9.46 (CH_3), 46.03 (*N*-CH_2), 112.13, 113.32, 119.15, 120.10, 121.44, 123.38, 123.97, 126.27, 129.13, 129.27, 132.80, 137.89, 142.54, 148.69, 152.77, 155.62, 157.95 (Aromatic carbons), 160.63 (C=O of hydrazide), 164.63 (C=O of isatin); IR (KBr, *ν* cm^{-1}) 3358 (NH), 2210 (CN) and 1724, 1700 (2C=O); MS *m*/*z* [%]: 434 [M^+, 73]; Anal. Calcd. for $C_{26}H_{18}N_4O_3$ (434.4): C, 71.88; H, 4.18; N, 12.90; found C, 71.80; H, 4.12; N, 12.94.

1.12. N'-(5-Bromo-2-oxo-1-propylindolin-3-ylidene)-3-methylbenzofuran-2-carbohydrazide ***7g***.

Yield 87%, m.p. 288-290 °C;^{1}H NMR *δ ppm*: 0.97 (t, 3H, -$CH_2C\underline{H}_3$, *J*= 8.0 Hz), 1.69-1.76 (m, 2H, -$C\underline{H}_2CH_3$), 2.72 (s, 3H, CH_3), 3.80 (t, 2H, *N*-CH_2, *J*= 8.0 Hz), 7.30 (d, 1H, Ar-H, *J*= 8.0 Hz), 7.46 (t, 1H, Ar-H, *J*= 8.0 Hz), 7.63 (t, 1H, Ar-H, *J*= 8.0 Hz), 7.71 (d, 1H, Ar-H, *J*= 8.0 Hz), 7.77-7.81(m, 2H, Ar-H), 7.90 (d, 1H, Ar-H, *J*= 8.0 Hz), 14.02 (s, 1H, NH, D_2O exchangeable);

^{13}C NMR δ *ppm*: 8.85 (CH_3), 13.00 (-$CH_2\underline{C}H_3$), 22.98 (*N*-CH_2-$\underline{C}H_2$), 48.74 (*N*-CH_2), 109.99, 113.17, 115.90, 119.28, 121.29, 124.38, 127.19, 129.37, 131.71, 134.92, 137.71, 143.11, 149.82, 151.50, 154.55 (Aromatic carbons), 161.36 (C=O of hydrazide), 167.02 (C=O of isatin); IR (KBr, ν cm^{-1}) 3335 (NH) and 1731, 1708 (2C=O); Anal. Calcd. for $C_{21}H_{18}BrN_3O_3$ (440.2): C, 57.29; H, 4.12; N, 9.54; found C, 57.21; H, 4.18; N, 9.50.

*1.13. N'-(1-Benzyl-5-bromo-2-oxoindolin-3-ylidene)-3-methylbenzofuran-2-carbohydrazide **7h**.*

Yield 79%, m.p. 214-216 °C; ^{1}H NMR δ *ppm*: 2.66 (s, 3H, CH_3), 5.05 (s, 2H, *N*-CH_2), 7.01 (d, 1H, Ar-H, *J*= 8.0 Hz), 7.29 (t, 1H, Ar-H, *J*= 8.0 Hz), 7.35 (t, 2H, Ar-H, *J*= 8.0 Hz), 7.41-7.44 (m, 3H, Ar-H), 7.55-7.60 (m, 2H, Ar-H), 7.69 (d, 1H, Ar-H, *J*= 8.0 Hz), 7.73 (s, 1H, Ar-H), 7.82 (d, 1H, Ar-H, *J*= 8.0 Hz), 13.91 (s, 1H, NH, D_2O exchangeable); ^{13}C NMR δ *ppm*: 8.12 (CH_3), 42.60 (*N*-CH_2), 106.76, 111.10, 115.71, 119.67, 121.59, 122.86, 124.39, 127.68, 129.39, 130.10, 134.89, 139.12, 140.40, 144.50, 148.48, 151.29, 153.71, 156.35, 157.61 (Aromatic carbons), 161.28 (C=O of hydrazide), 166.40 (C=O of isatin); IR (KBr, ν cm^{-1}) 3240 (NH) and 1730, 1711 (2C=O); Anal. Calcd. for $C_{25}H_{18}BrN_3O_3$ (488.3): C, 61.49; H, 3.72; N, 8.60; found C, 61.40; H, 3.77; N, 8.65.

*1.14. N'-(5-Bromo-1-(4-fluorobenzyl)-2-oxoindolin-3-ylidene)-3-methylbenzofuran-2-carbohydrazide **7i**.*

Yield 75%, m.p. 238-240 °C; ^{1}H NMR δ *ppm*: 2.68 (s, 3H, CH_3), 5.06 (s, 2H, *N*-CH_2), 7.06 (d, 1H, Ar-H, *J*= 8.0 Hz), 7.18 (t, 2H, Ar-H, *J*= 8.0 Hz), 7.42 (t, 1H, Ar-H, *J*= 8.0 Hz), 7.48 (t, 2H, Ar-H, *J*= 8.0 Hz), 7.5-7.64 (m, 2H, Ar-H), 7.72 (d, 1H, Ar-H, *J*= 8.0 Hz), 7.78 (s, 1H, Ar-H), 7.86 (d, 1H, Ar-H, *J*= 8.0 Hz), 13.94 (s, 1H, NH, D_2O exchangeable); IR (KBr, ν cm^{-1}) 3270 (NH) and 1728, 1707 (2C=O); Anal. Calcd. for $C_{25}H_{17}BrFN_3O_3$ (506.3): C, 59.30; H, 3.38; N, 8.30; found C, 59.21; H, 3.31; N, 8.36.

2. Biological Evaluation

2.1. NCI, USA Anticancer Screening

The cytotoxicity *in vitro* assay was conducted at National Cancer Institute (NCI), Bethesda, USA against 59 cancer cell lines. The one-dose data were reported as a mean graph of the percent growth of treated cells. The number reported for the one-dose assay was growth relative to the no-drug control and relative to the time zero number of cells. This allowed detection of both growth inhibition (values between 0 and 100) and lethality (values less than 0). For example, a value of 100 means no growth inhibition. A value of 40 would mean 60% growth inhibition. A value of 0 means no net growth over the course of the experiment. A value of -40 would mean 40% lethality. A value of -100 means all cells were dead.

The human tumor cell lines of the cancer screening panel were grown in RPMI 1640 medium containing 5% fetal bovine serum and 2 mM L-glutamine. For a typical screening experiment, cells were inoculated into 96 well microtiter plates in 100 µL at plating densities ranging from 5,000 to 40,000 cells/well depending on the doubling time of individual cell lines. After cell inoculation, the microtiter plates were incubated at 37° C, 5 % CO2, 95 % air and 100 % relative humidity for 24 h prior to addition of experimental drugs. After 24 h, two plates of each cell line were fixed *in situ* with TCA, to represent a measurement of the cell population for each cell line at the time of drug addition (Tz). Experimental drugs were solubilized in dimethyl sulfoxide at 400-fold the desired final maximum test concentration and stored frozen prior to use. At the time of drug addition, an aliquot of frozen concentrate was thawed and diluted to twice the desired final maximum test concentration with complete medium containing 50 µg/mL gentamicin. Additional four, 10-fold or ½ log serial dilutions were made to provide a total of five drug concentrations plus control. Aliquots of 100 µL of these different drug dilutions were added to the appropriate microtiter wells already containing 100 µL of medium, resulting in the required final drug concentrations. Following drug addition, the plates were incubated for an additional 48 h at 37°C, 5 % CO2, 95 % air, and 100 % relative humidity. For adherent cells, the assay was terminated by the addition of cold TCA. Cells were fixed *in situ* by the gentle addition of 50 µL of cold 50 % (w/v) TCA (final concentration, 10 % TCA) and incubated for 60 minutes at 4°C. The supernatant was discarded, and the plates were washed five times with tap water and air dried. Sulforhodamine B (SRB) solution (100 µL) at 0.4 % (w/v) in 1 % acetic acid was added to

each well, and plates were incubated for 10 minutes at room temperature. After staining, unbound dye was removed by washing five times with 1 % acetic acid and the plates were air dried. Bound stain was subsequently solubilized with 10 mM trizma base, and the absorbance was read on an automated plate reader at a wavelength of 515 nm. For suspension cells, the methodology was the same except that the assay was terminated by fixing settled cells at the bottom of the wells by gently adding 50 µL of 80 % TCA (final concentration, 16 % TCA). Using the seven absorbance measurements [time zero, (Tz), control growth, (C), and test growth in the presence of drug at the five concentration levels (Ti)], the percentage growth was calculated at each of the drug concentrations levels. Percentage growth inhibition was calculated as:

[(Ti-Tz)/(C-Tz)] x 100 for concentrations for which Ti>/=Tz

[(Ti-Tz)/Tz] x 100 for concentrations for which Ti<Tz

Three dose response parameters were calculated for each experimental agent. Growth inhibition of 50 % (GI_{50}) was calculated from [(Ti-Tz)/(C-Tz)] x 100 = 50, which is the drug concentration resulting in a 50% reduction in the net protein increase (as measured by SRB staining) in control cells during the drug incubation. The drug concentration resulting in total growth inhibition (TGI) was calculated from Ti = Tz. The LC_{50} (concentration of drug resulting in a 50% reduction in the measured protein at the end of the drug treatment as compared to that at the beginning) indicating a net loss of cells following treatment was calculated from [(Ti-Tz)/Tz] x 100 = -50.

2.2. Cell Culture

SW620 and HT29 colorectal cancer cell lines and HFF-1 fibroblast (ATCC, Rockville, USA) were used. HT29 cells were cultured and maintained in Dulbecco's Modified Eagles Medium (DMEM) (GIBCO, by Thermo Fischer Scientific, NY, USA) supplemented with 10% fetal bovine serum (FBS), 100 units/mL penicillin, and 100 µg streptomycin. SW620 and HFF-1 cells were cultured in Roswell Park Memorial Institute medium (RPMI-1640) (GIBCO, by Thermo scientific, NY, USA) supplemented with 10% FBS and 1% penicillin and streptomycin (Napolitano et al., 2015). All cultures were incubated at 37 °C and humidified atmosphere of 5% CO_2.

2.3. Cell Viability Assay

The cytotoxicity effect of compounds on the colorectal cancer cell lines, SW620 and HT29 in addition to the normal human fibroblasts was measured by MTT (3-(4, 5-dimethylthiazol-2-yl)-2,5-diphenyltetrazolium bromide) (Sigma-Aldrich, St. Louis, MO, USA) as previously described [39]. Briefly, cells were seeded in 96 well culture plates at 5000/well for HT29 and 10,000/well for SW620 for 24 h. Cells were then incubated with different compounds from WAG1 series (1-15) 24h at 37 °C and humidified 5% CO_2 incubator. Freshly prepared 10 µl of 3-(4,5-dimethylthiazol-2-yl)-2,5-diphenyltetrazolium bromide (MTT 5 mM) solution were added to the cells and further incubated for 2 h. Thereafter, 100 µl of dimethyl sulfoxide (DMSO) were added in each well and the crystals were dissolved through careful pipetting. In certain experiment, cells were treated different concentration of 5FU for 72 h. The absorbance of the product was measured at 540 nm using a Synergy™ 2 multi-mode microplate reader (Biotech, VA, USA). The experiments were performed in triplicate for each condition.

2.4. Measurement of Apoptosis by Annexin V-FITC/PI Assay

Induction of apoptosis was measured by Dead Cell Apoptosis Kit with Annexin V FITC and PI, for flow cytometry (Thermofischer scientific, OR, USA) according to the manufacturer's instruction. SW620 cells were seeded in a 6-well plate (3×10^5 cells per well) and treated with the various compound for 24h. Both floating and adherent cells were harvested, pooled together, and incubated with Annexin V-FITC and PI for 15 min on ice in dark. The cells were analyzed by BD FACSCalibur™ cell analyzer (BD Biosciences, CA, USA) at an emission of 530 nm (FL1 channel) and >575 nm (FL3).

2.5. Western Blot Analysis

All cells were seeded in a 100 mm dish (1×10^6 cells per dish) in 5% CO_2 at 37 °C in the appropriate culture medium. The cells with around 50% confluency were treated with compounds for 24h. At the experiment day, cells were washed with 1x PBS, harvested and lysed in RIPA lysis buffer, combined with protease inhibitors, (Sigma-Aldrich, St. Louis, MO, USA) as described previously (39)). The total protein concentration was evaluated by the colorimetric Bradford protein assay (BIO-RAD inc, CA, USA) at 595 nm absorbance. Lysates were loaded in

equal concentration and separated by sodium dodecyl sulfate-polyacrylamide gel electrophoresis (SDS-PAGE) and then transferred to a nitrocellulose membrane by semi-dry. Blocking of the membrane was done by 5% non-fat dried milk for one hour, incubated with the primary antibodies. The primary antibodies used were Bcl2 (25 kDa, cat. no. sc-7382), and β Actin (43 kDa, cat. no. sc-69879) from (Santa Cruz Biotechnology, Inc., Dallas, TX, USA). The secondary antibodies used were goat anti-mouse IgG-HRP (cat. no. sc-2005) and mouse anti-rabbit IgG-HRP (cat. no. sc-2357) from (Santa Cruz Biotechnology, Inc., Dallas, TX, USA). Detection was done with Luminol HRP chemiluminescence substrate (cat. no. sc-2048) from (Santa Cruz Biotechnology, Inc., Dallas, TX, USA) and then visualized by c-digit blot-scanner (LI-COR, Nebraska, USA).

2.6. Statistical Analysis

The statistical analysis by the One-way ANOVA test was performed by GraphPad prism. Results were considered significant if the *P-Values* were <0.05.

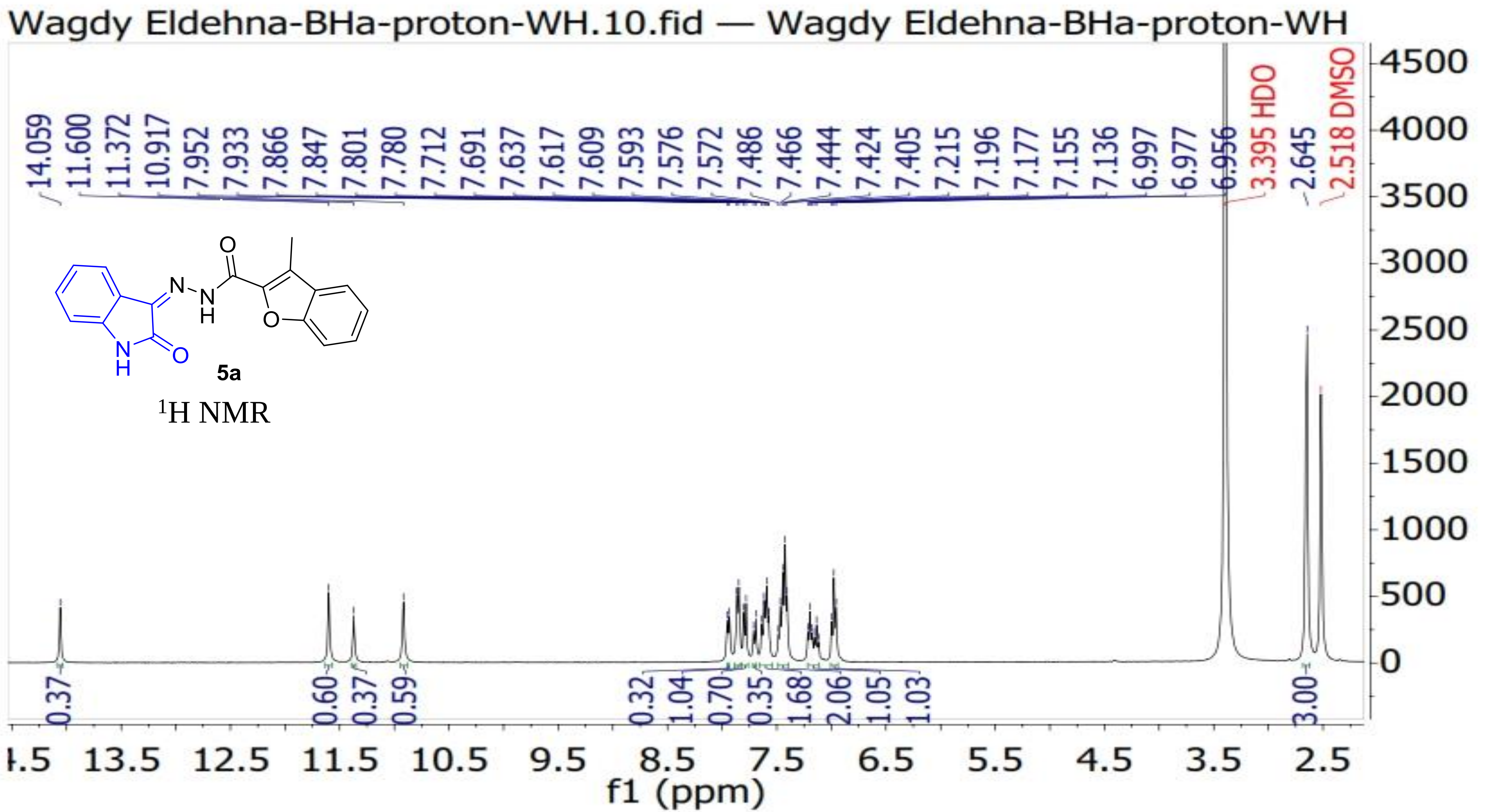
Wagdy Eldehna-BHa-proton-WH.10.fid — Wagdy Eldehna-BHa-proton-WH
14.059
11.600
11.372
10.917
7.952
7.933
7.866
7.847
7.801
7.780
7.712
7.691
7.637
7.617
7.609
7.593
7.576
7.572
7.486
7.466
7.444
7.424
7.405
7.215
7.196
7.177
7.155
7.136
6.997
6.977
6.956
3.395 HDO
2.645
2.518 DMSO
5a
1H NMR
0.37
0.60
0.37
0.59
0.32
1.04
0.70
0.35
1.68
2.06
1.05
1.03
3.00
f1 (ppm)

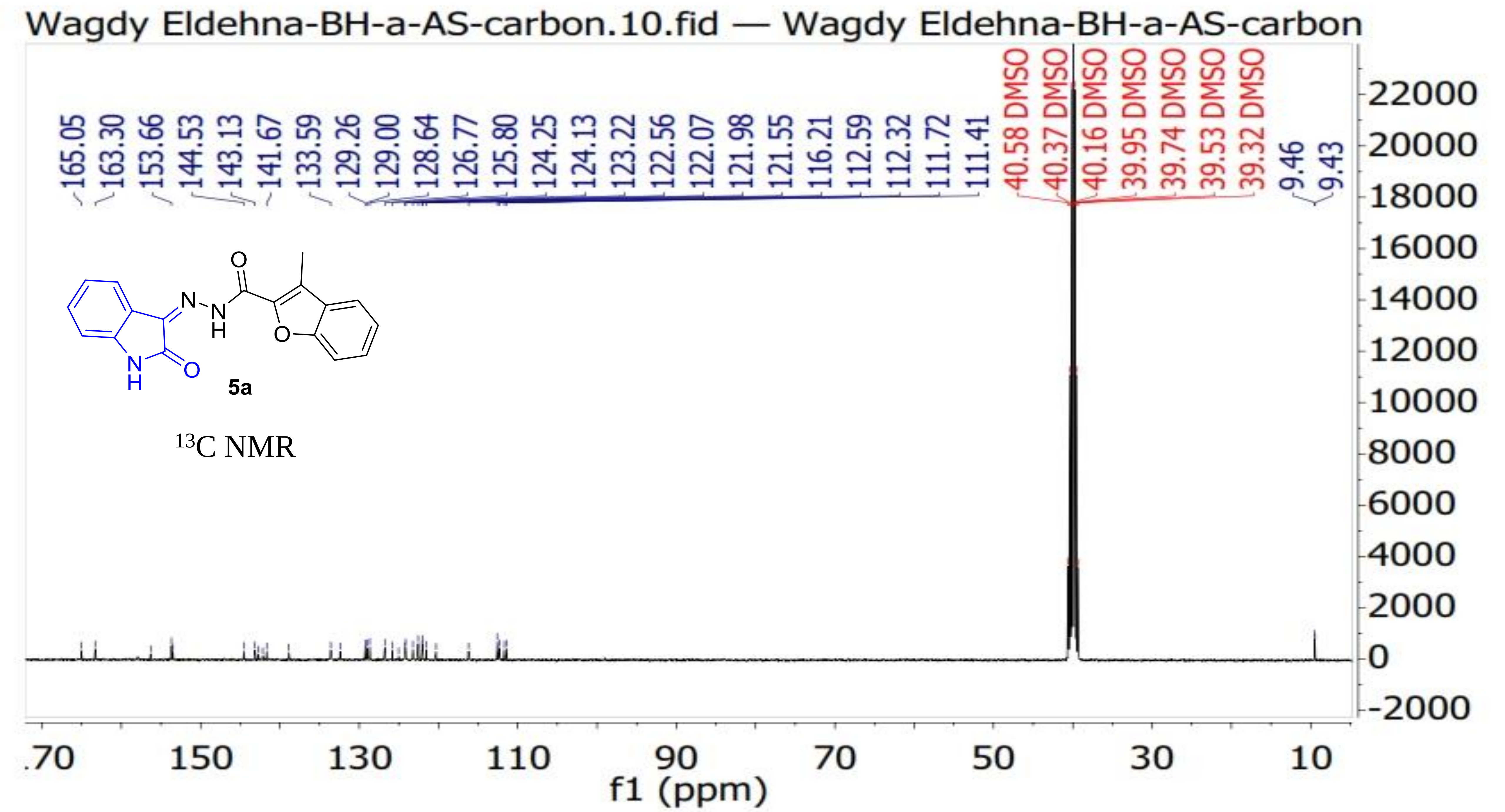
Wagdy Eldehna-BH-a-AS-carbon.10.fid — Wagdy Eldehna-BH-a-AS-carbon
165.05
163.30
153.66
144.53
143.13
141.67
133.59
129.26
129.00
128.64
126.77
125.80
124.25
124.13
123.22
122.56
122.07
121.98
121.55
116.21
112.59
112.32
111.72
111.41
40.58 DMSO
40.37 DMSO
40.16 DMSO
39.95 DMSO
39.74 DMSO
39.53 DMSO
39.32 DMSO
9.46
9.43
O
N
N
H
O
N
H
O
5a
13C NMR
22000
20000
18000
16000
14000
12000
10000
8000
6000
4000
2000
0
-2000
.70
150
130
110
90
70
50
30
10
f1 (ppm)

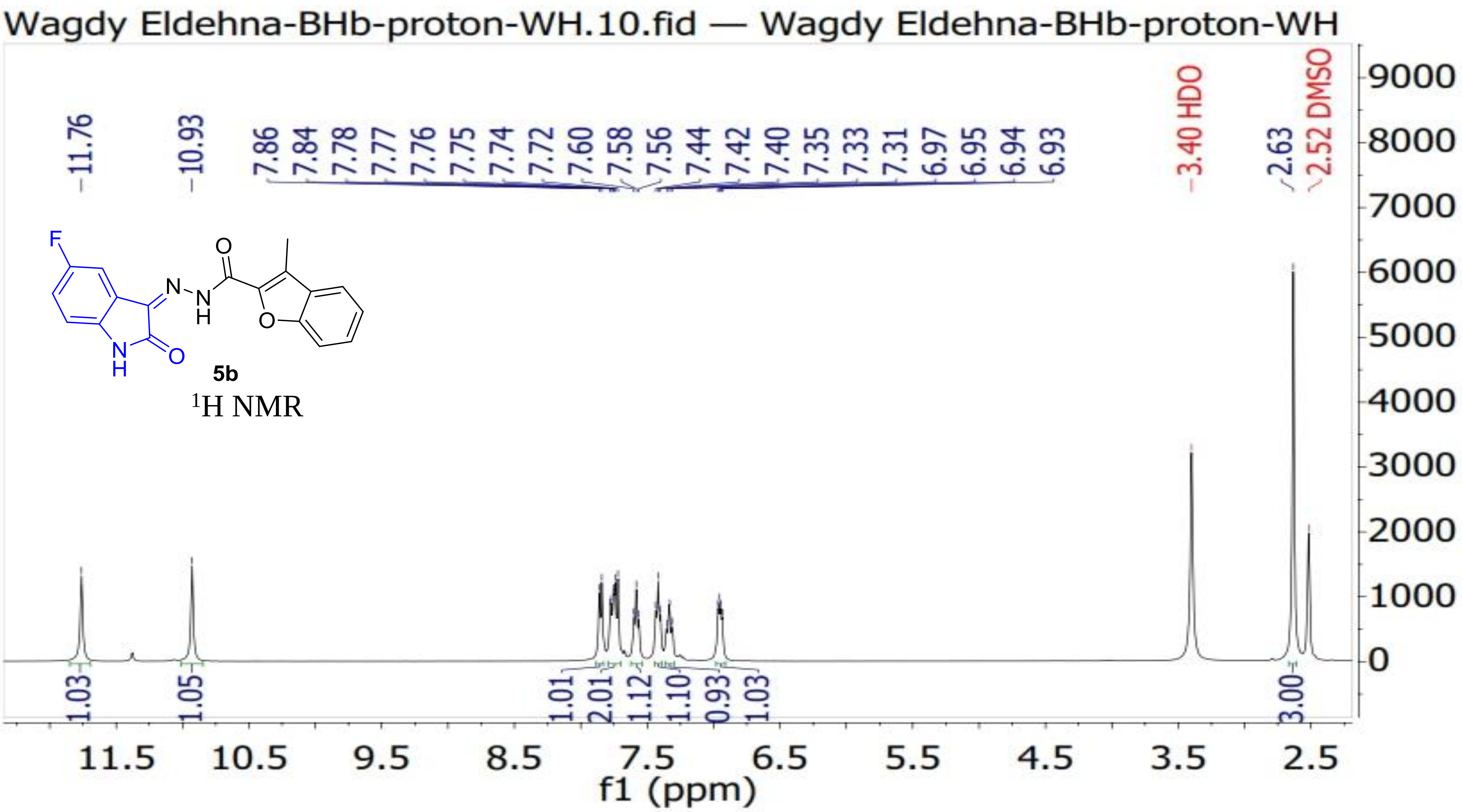

Wagdy Eldehna-BHb-proton-WH.10.fid — Wagdy Eldehna-BHb-proton-WH
11.76
10.93
7.86 7.84 7.78 7.77 7.76 7.75 7.74 7.72 7.60 7.58 7.56 7.44 7.42 7.40 7.35 7.33 7.31 6.97 6.95 6.94 6.93
3.40 HDO
2.63
2.52 DMSO
F
N
N
H
O
O
NH
O
5b
1H NMR
1.03
1.05
1.01 2.01 1.12 1.10 0.93 1.03
3.00
f1 (ppm)

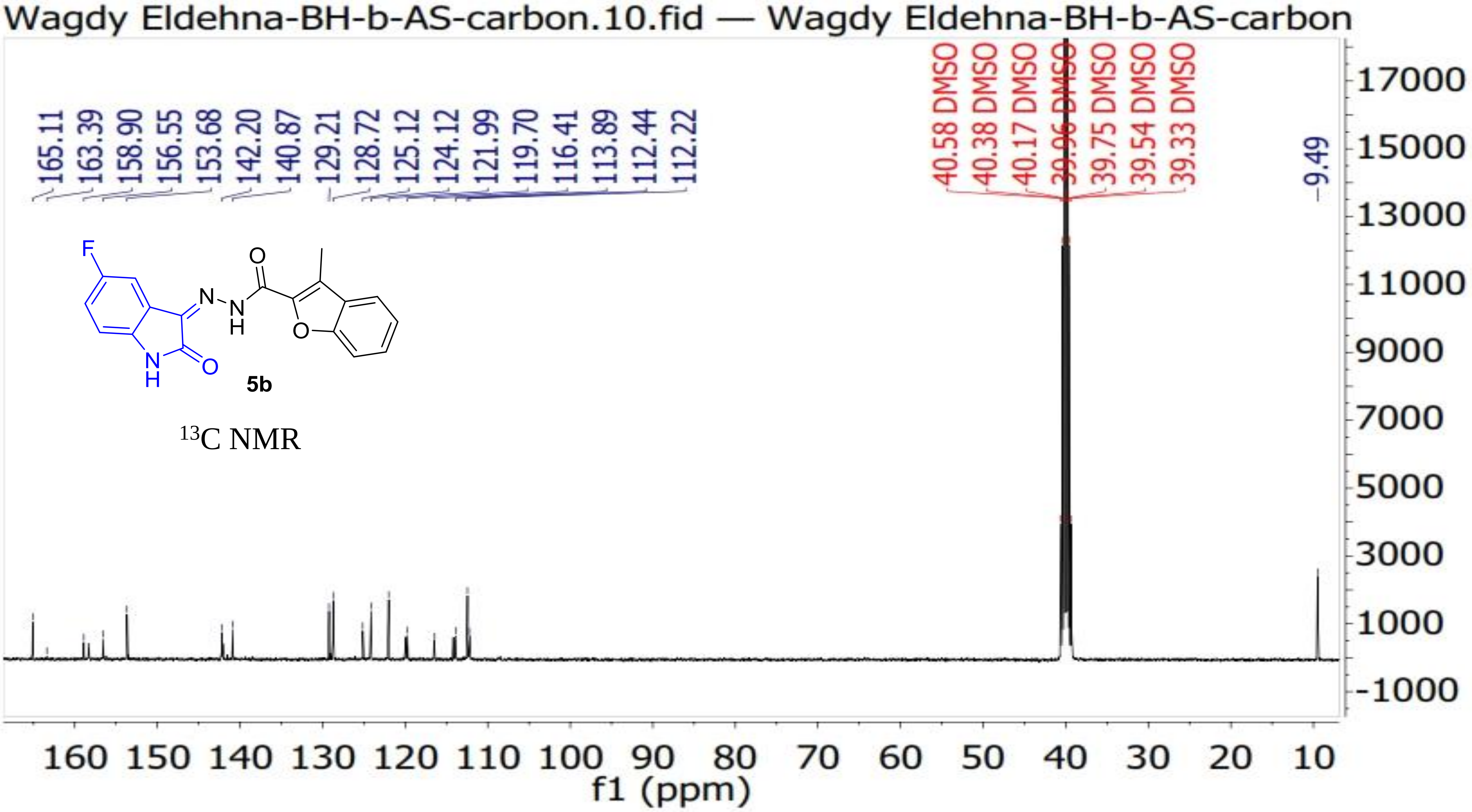
Wagdy Eldehna-BH-b-AS-carbon.10.fid — Wagdy Eldehna-BH-b-AS-carbon
165.11
163.39
158.90
156.55
153.68
142.20
140.87
129.21
128.72
125.12
124.12
121.99
119.70
116.41
113.89
112.44
112.22
40.58 DMSO
40.38 DMSO
40.17 DMSO
39.96 DMSO
39.75 DMSO
39.54 DMSO
39.33 DMSO
9.49
F
O
N
N
H
O
NH
O
5b
^{13}C NMR
f1 (ppm)

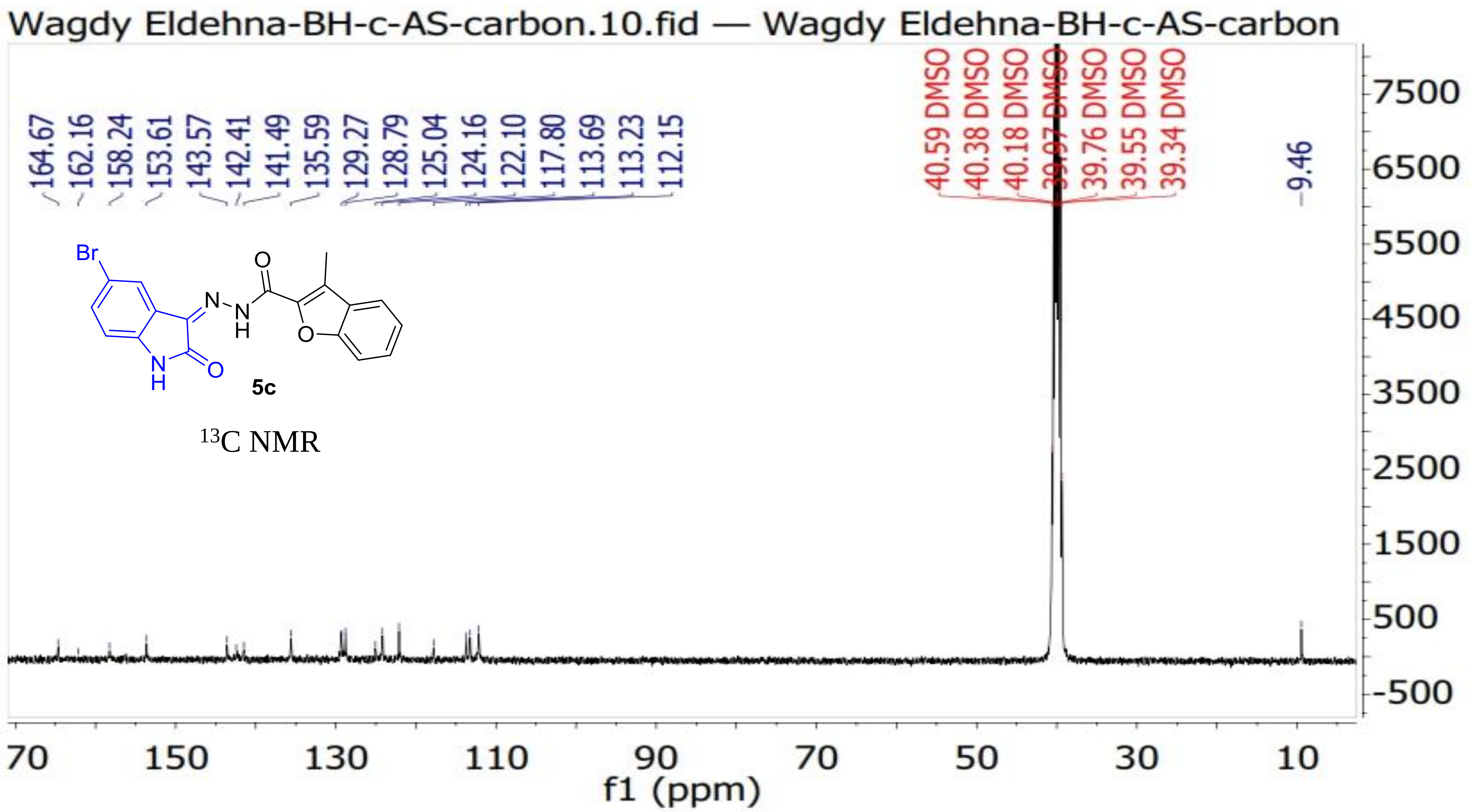
Wagdy Eldehna-BH-c-AS-carbon.10.fid — Wagdy Eldehna-BH-c-AS-carbon
164.67
162.16
158.24
153.61
143.57
142.41
141.49
135.59
129.27
128.79
125.04
124.16
122.10
117.80
113.69
113.23
112.15
40.59 DMSO
40.38 DMSO
40.18 DMSO
39.97 DMSO
39.76 DMSO
39.55 DMSO
39.34 DMSO
9.46
Br
O
N
N
H
O
NH
O
5c
13C NMR
7500
6500
5500
4500
3500
2500
1500
500
-500
70
150
130
110
90
70
50
30
10
f1 (ppm)

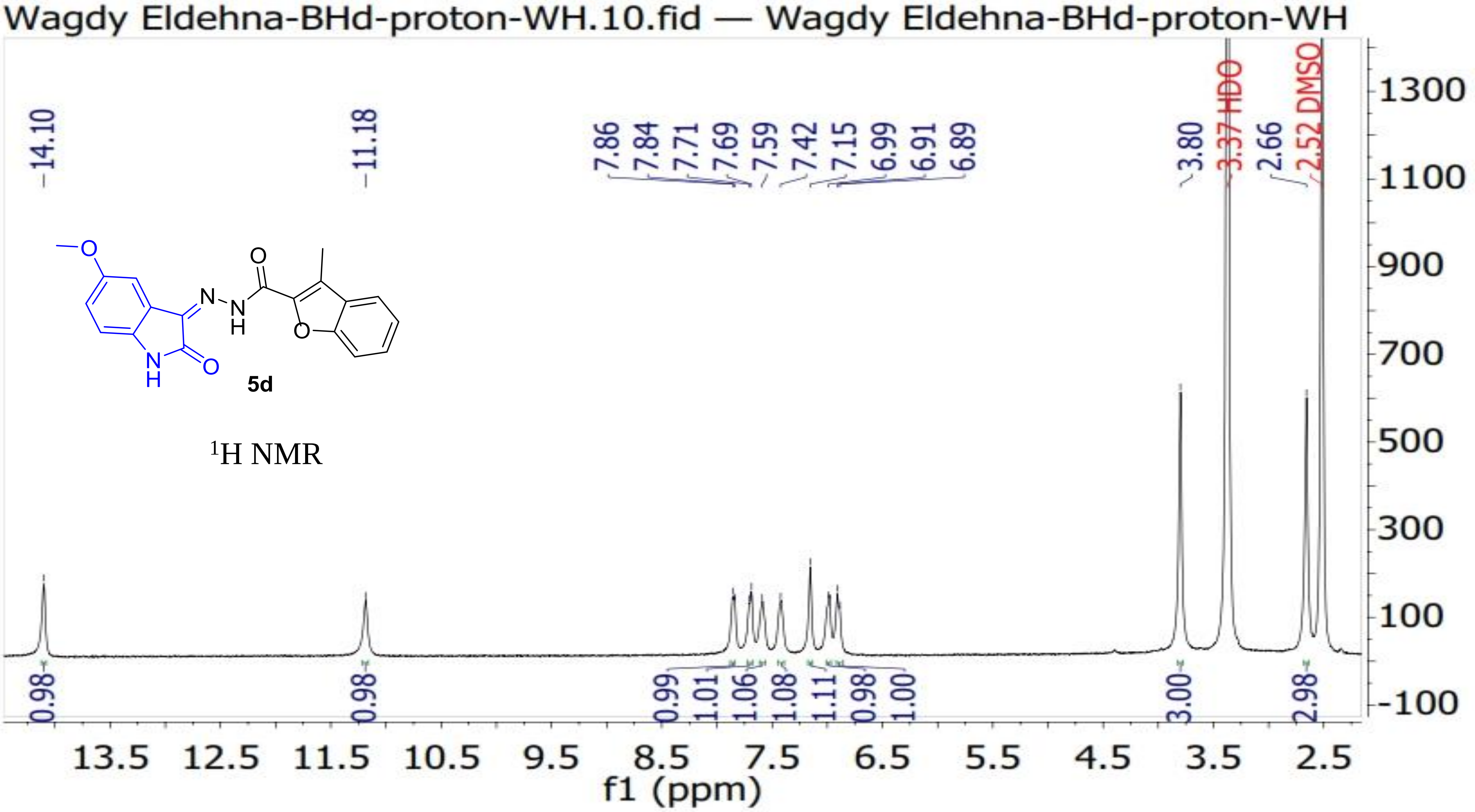

Wagdy Eldehna-BHd-proton-WH.10.fid — Wagdy Eldehna-BHd-proton-WH
14.10
11.18
7.86
7.84
7.71
7.69
7.59
7.42
7.15
6.99
6.91
6.89
3.80
3.37 HDO
2.66
2.52 DMSO
O
N
N
H
O
O
NH
O
5d
1H NMR
0.98
0.98
0.99
1.01
1.06
1.08
1.11
0.98
1.00
3.00
2.98
13.5
12.5
11.5
10.5
9.5
8.5
7.5
6.5
5.5
4.5
3.5
2.5
f1 (ppm)
1300
1100
900
700
500
300
100
-100

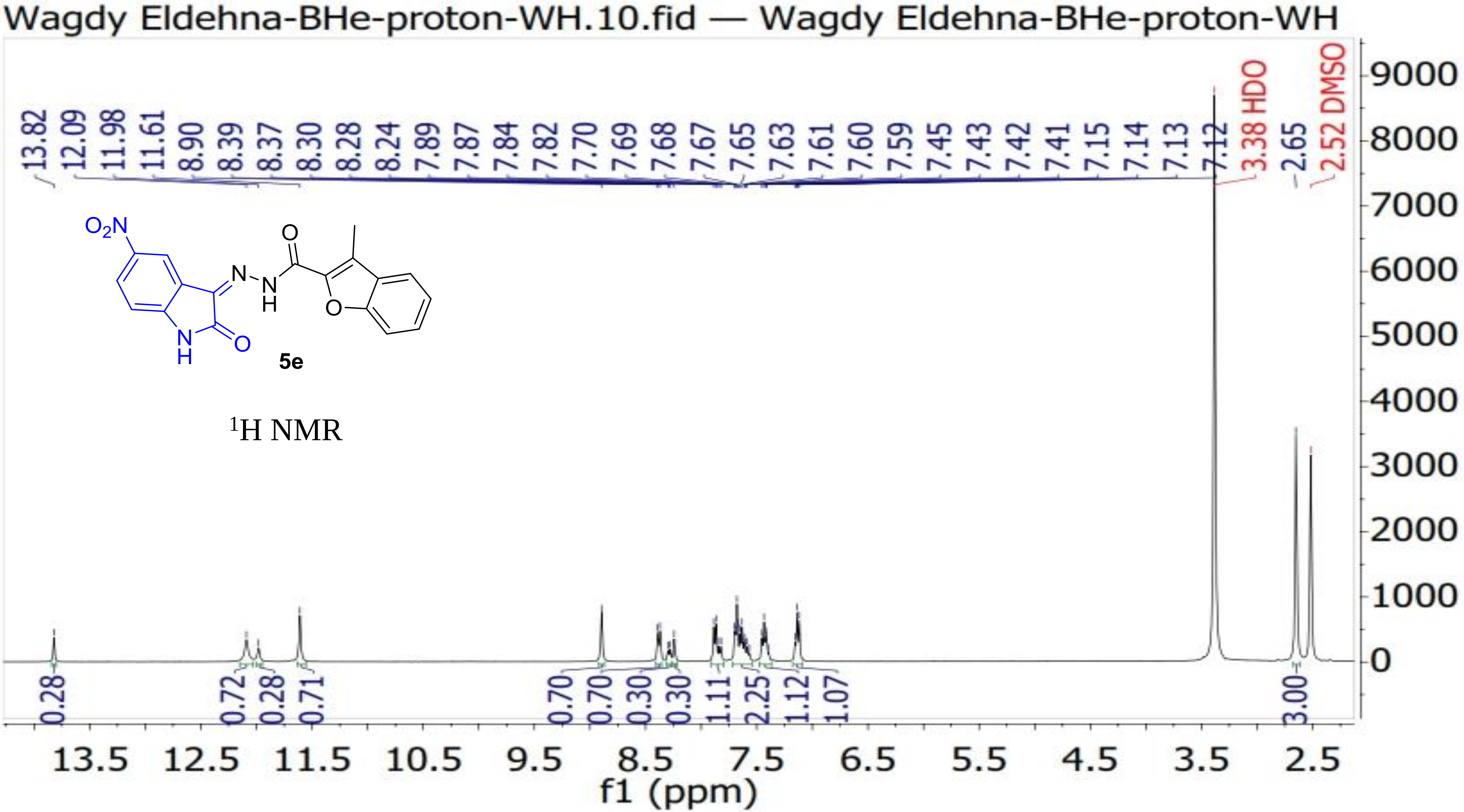
Wagdy Eldehna-BHe-proton-WH.10.fid — Wagdy Eldehna-BHe-proton-WH
13.82
12.09
11.98
11.61
8.90
8.39
8.37
8.30
8.28
8.24
7.89
7.87
7.84
7.82
7.70
7.69
7.68
7.67
7.65
7.63
7.61
7.60
7.59
7.45
7.43
7.42
7.41
7.15
7.14
7.13
7.12
3.38 HDO
2.65
2.52 DMSO
O2N
O
N
N
H
NH
O
O
H
5e
1H NMR
0.28
0.72
0.28
0.71
0.70
0.70
0.30
0.30
1.11
2.25
1.12
1.07
3.00
9000
8000
7000
6000
5000
4000
3000
2000
1000
0
13.5
12.5
11.5
10.5
9.5
8.5
7.5
6.5
5.5
4.5
3.5
2.5
f1 (ppm)

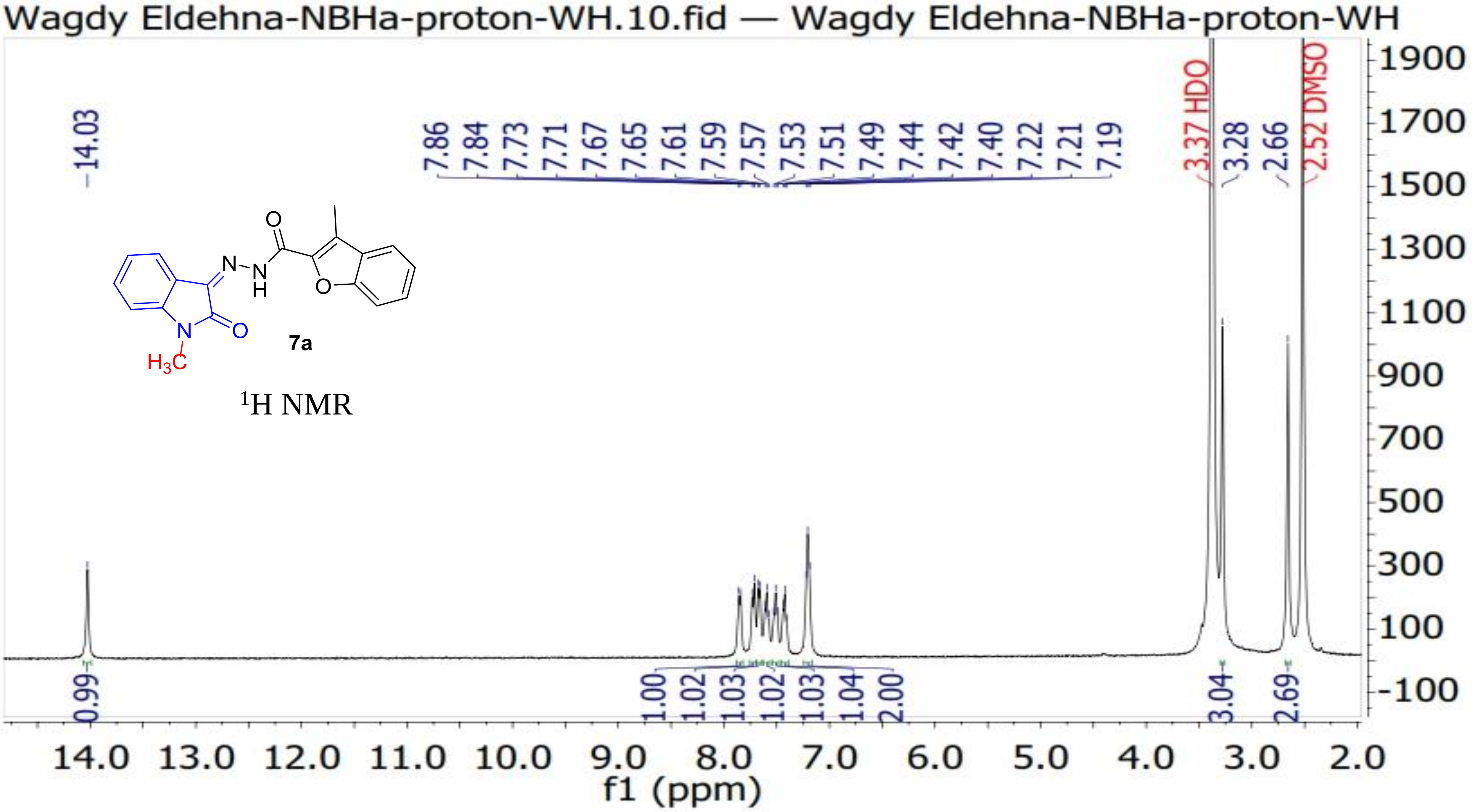

Wagdy Eldehna-NBHa-proton-WH.10.fid — Wagdy Eldehna-NBHa-proton-WH
14.03
7.86
7.84
7.73
7.71
7.67
7.65
7.61
7.59
7.57
7.53
7.51
7.49
7.44
7.42
7.40
7.22
7.21
7.19
3.37 HDO
3.28
2.66
2.52 DMSO
O
N
N
H
O
N
O
H3C
7a
1H NMR
0.99
1.00
1.02
1.03
1.02
1.03
1.04
2.00
3.04
2.69
1900
1700
1500
1300
1100
900
700
500
300
100
-100
14.0
13.0
12.0
11.0
10.0
9.0
8.0
7.0
6.0
5.0
4.0
3.0
2.0
f1 (ppm)

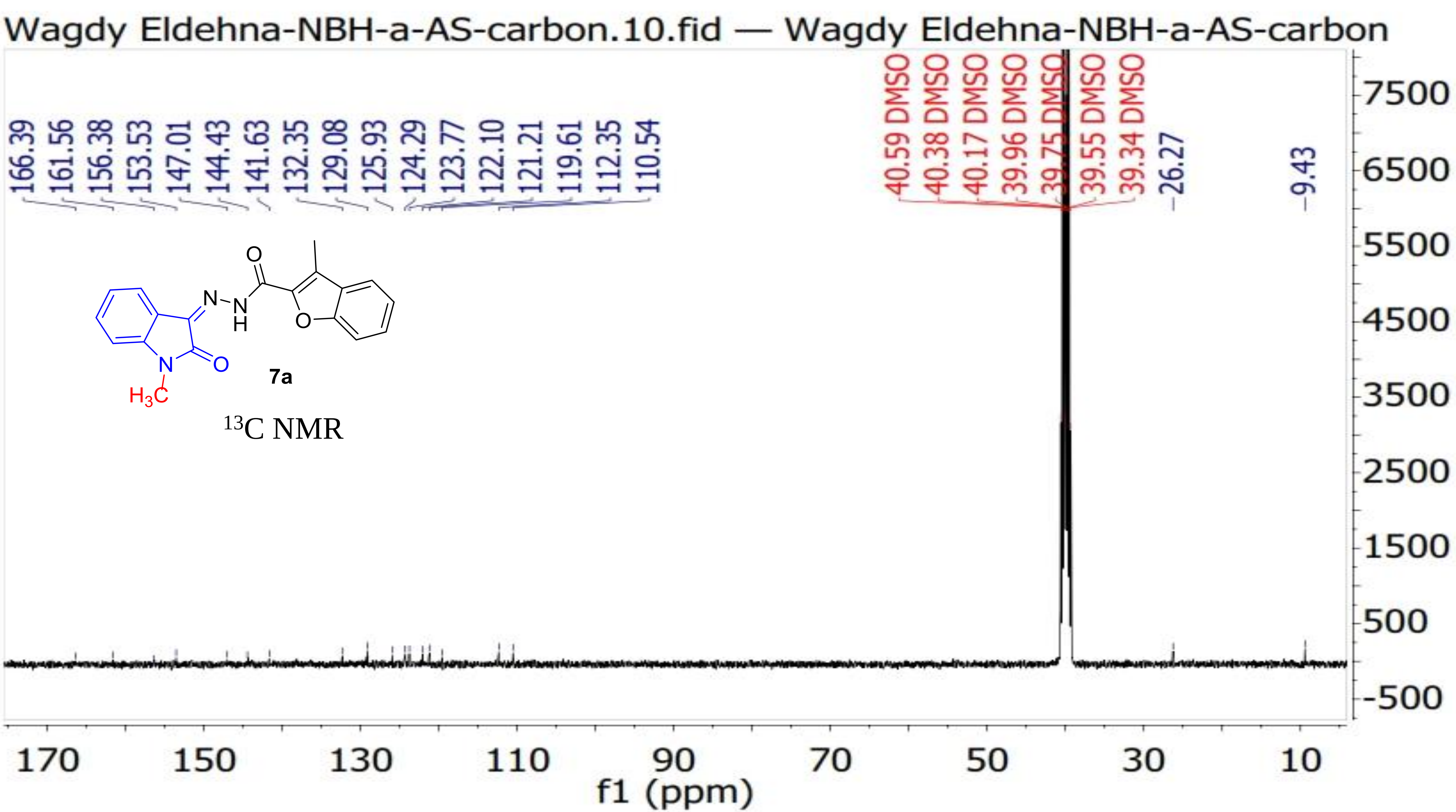

Wagdy Eldehna-NBH-a-AS-carbon.10.fid — Wagdy Eldehna-NBH-a-AS-carbon
166.39
161.56
156.38
153.53
147.01
144.43
141.63
132.35
129.08
125.93
124.29
123.77
122.10
121.21
119.61
112.35
110.54
40.59 DMSO
40.38 DMSO
40.17 DMSO
39.96 DMSO
39.75 DMSO
39.55 DMSO
39.34 DMSO
26.27
9.43
O
N
N
H
O
N
O
H_3C
7a
^{13}C NMR
f1 (ppm)

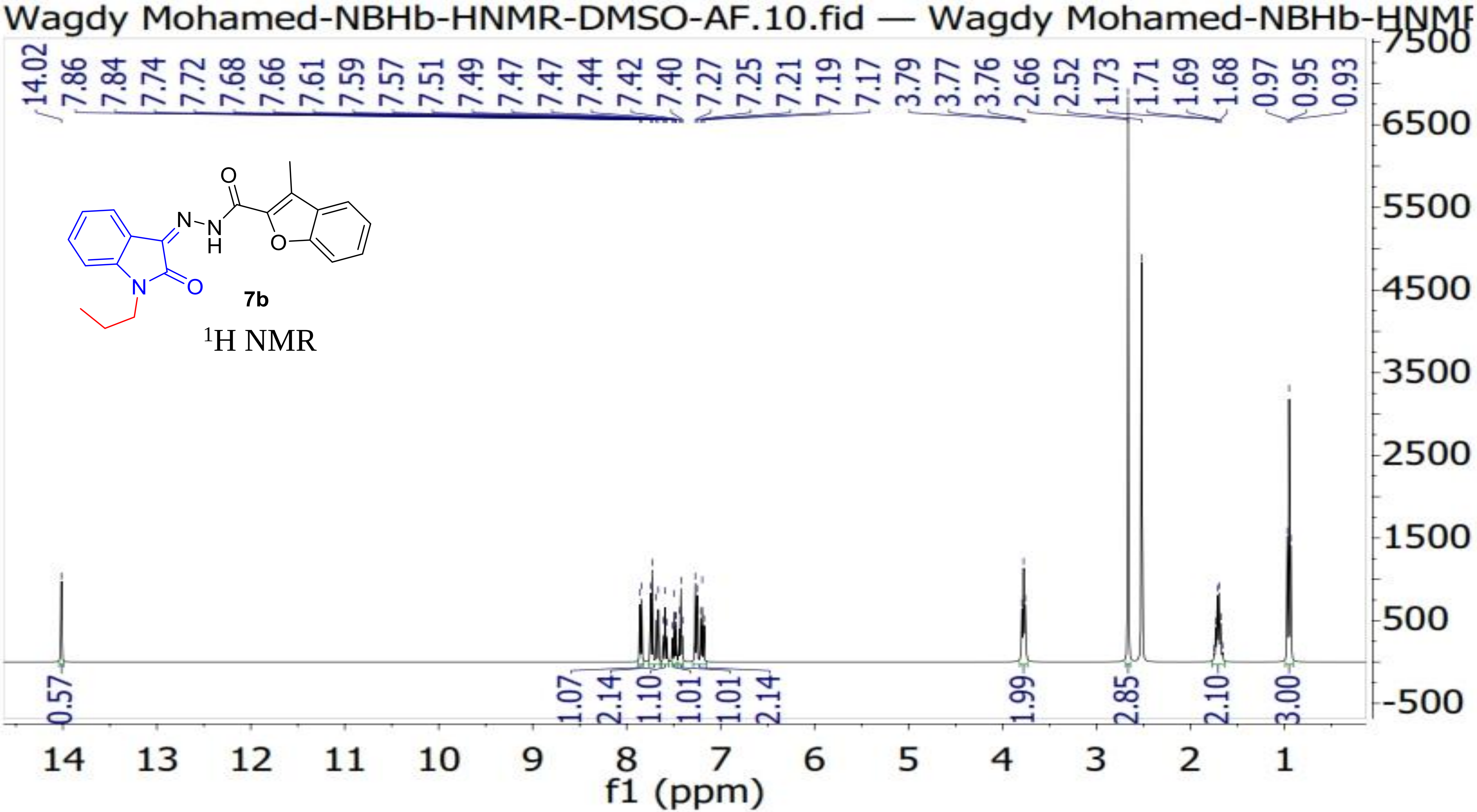

Wagdy Mohamed-NBHb-HNMR-DMSO-AF.10.fid — Wagdy Mohamed-NBHb-HNMI
14.02
7.86
7.84
7.74
7.72
7.68
7.66
7.61
7.59
7.57
7.51
7.49
7.47
7.47
7.44
7.42
7.40
7.27
7.25
7.21
7.19
7.17
3.79
3.77
3.76
2.66
2.52
1.73
1.71
1.69
1.68
0.97
0.95
0.93
7b
1H NMR
0.57
1.07
2.14
1.10
1.01
1.01
2.14
1.99
2.85
2.10
3.00
f1 (ppm)

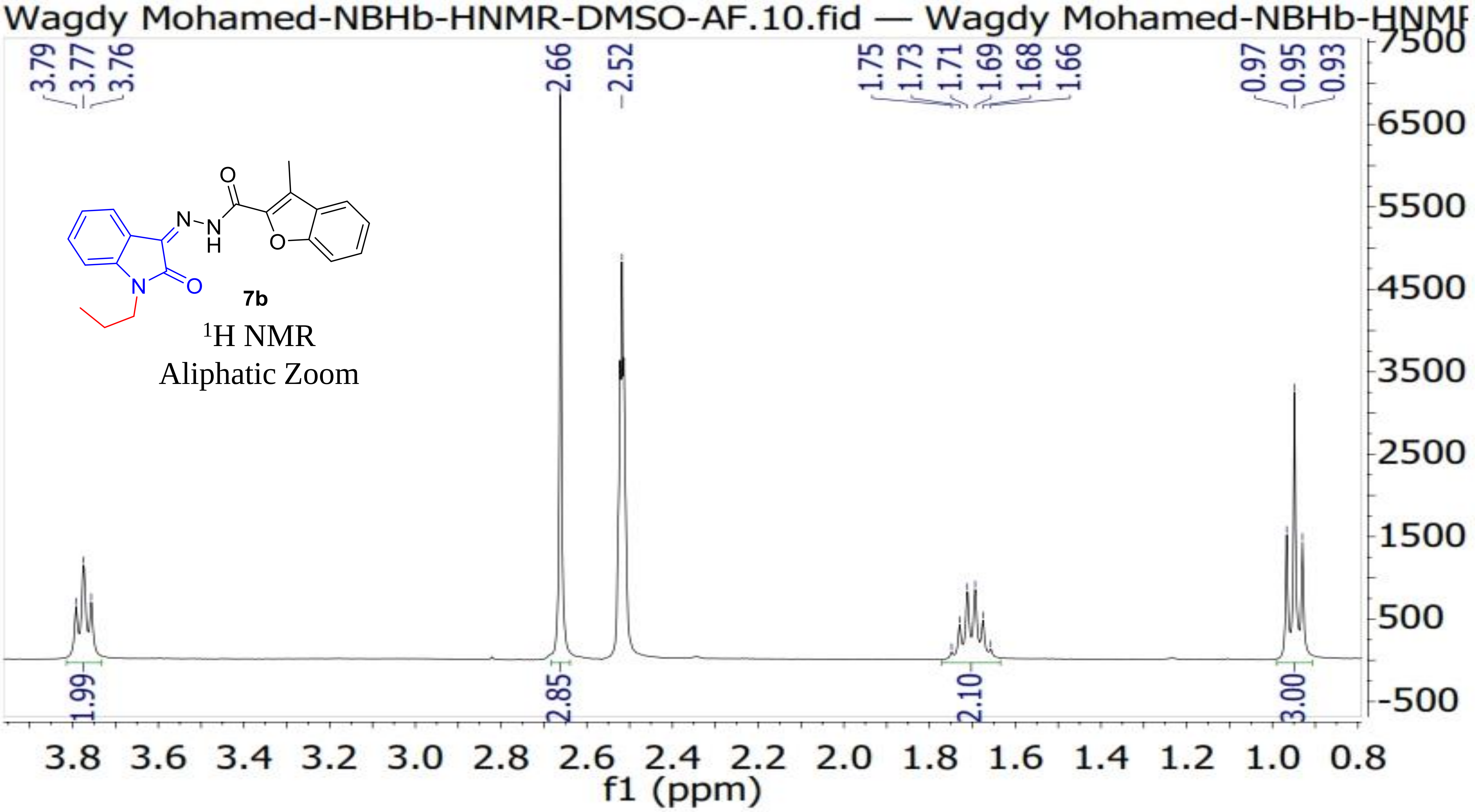
Wagdy Mohamed-NBHb-HNMR-DMSO-AF.10.fid — Wagdy Mohamed-NBHb-HNMR
3.79
3.77
3.76
2.66
2.52
1.75
1.73
1.71
1.69
1.68
1.66
0.97
0.95
0.93
7b
1H NMR
Aliphatic Zoom
1.99
2.85
2.10
3.00
f1 (ppm)

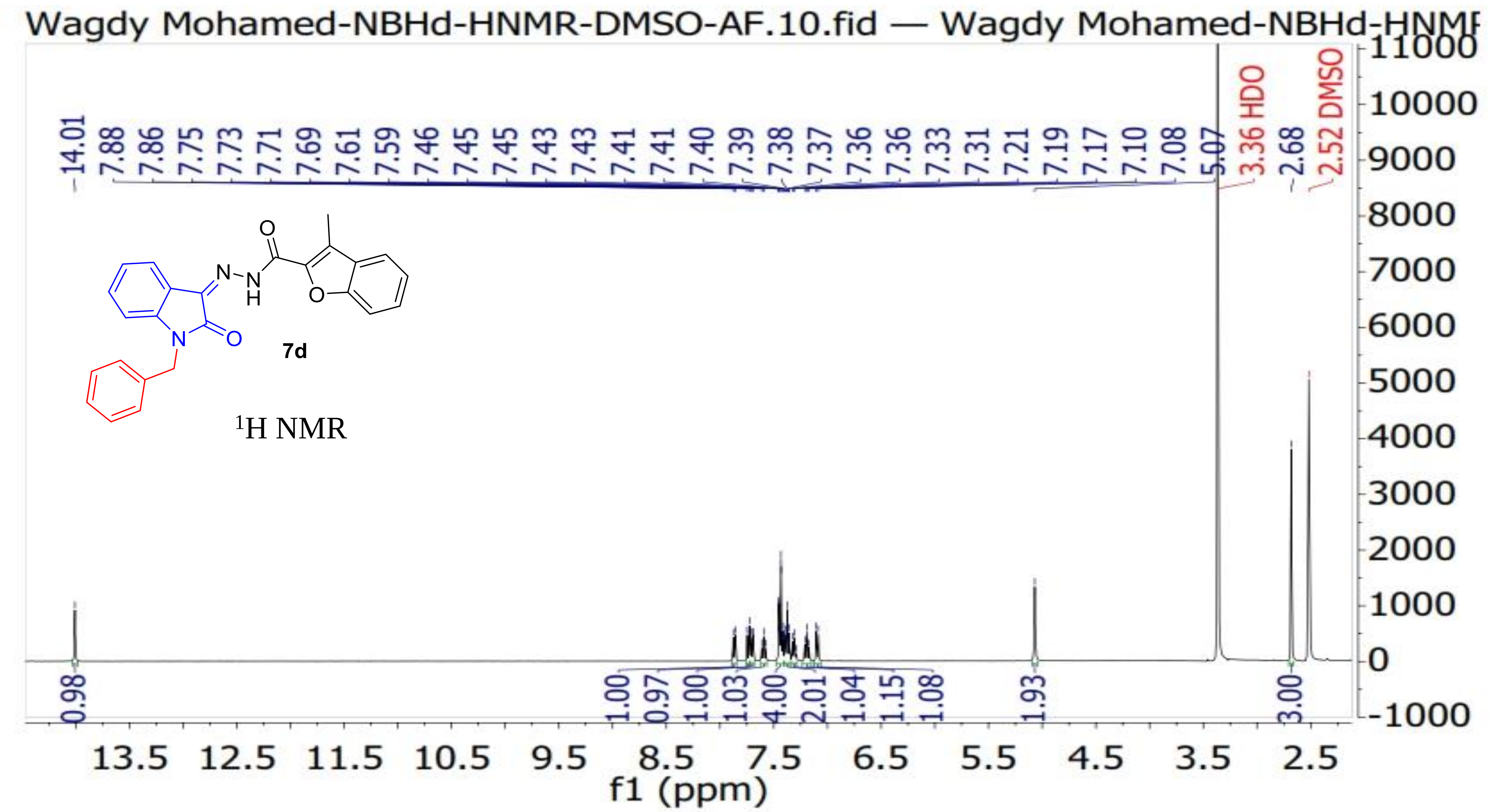
Wagdy Mohamed-NBHd-HNMR-DMSO-AF.10.fid — Wagdy Mohamed-NBHd-HNMI
14.01
7.88
7.86
7.75
7.73
7.71
7.69
7.61
7.59
7.46
7.45
7.45
7.43
7.43
7.41
7.41
7.40
7.39
7.38
7.37
7.36
7.36
7.33
7.31
7.21
7.19
7.17
7.10
7.08
5.07
3.36 HDO
2.68
2.52 DMSO
O
N
N
H
O
N
O
7d
1H NMR
0.98
1.00
0.97
1.00
1.03
4.00
2.01
1.04
1.15
1.08
1.93
3.00
11000
10000
9000
8000
7000
6000
5000
4000
3000
2000
1000
0
-1000
13.5
12.5
11.5
10.5
9.5
8.5
7.5
6.5
5.5
4.5
3.5
2.5
f1 (ppm)

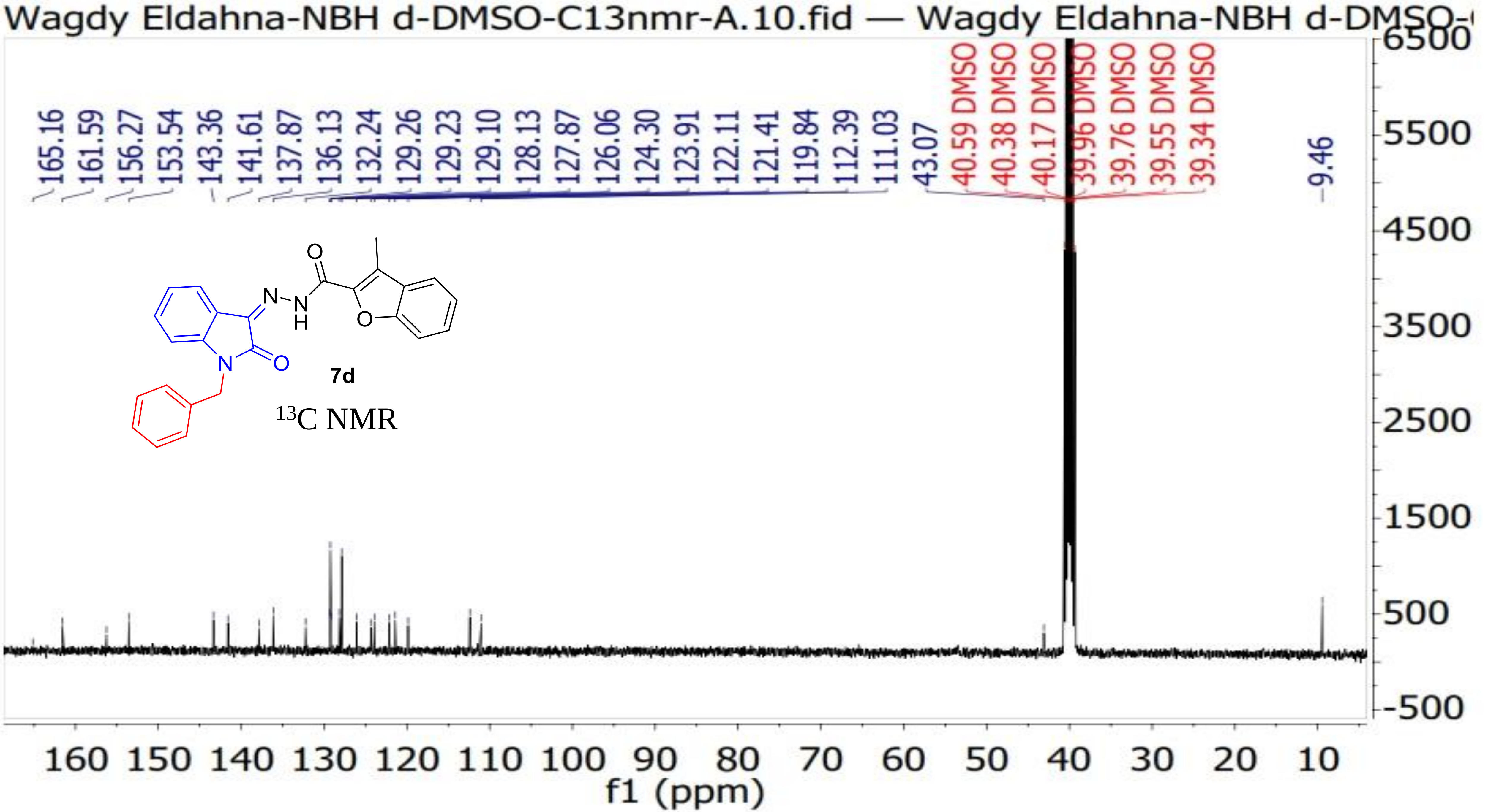
Wagdy Eldahna-NBH d-DMSO-C13nmr-A.10.fid — Wagdy Eldahna-NBH d-DMSO-
165.16
161.59
156.27
153.54
143.36
141.61
137.87
136.13
132.24
129.26
129.23
129.10
128.13
127.87
126.06
124.30
123.91
122.11
121.41
119.84
112.39
111.03
43.07
40.59 DMSO
40.38 DMSO
40.17 DMSO
39.96 DMSO
39.76 DMSO
39.55 DMSO
39.34 DMSO
9.46
7d
13C NMR
f1 (ppm)

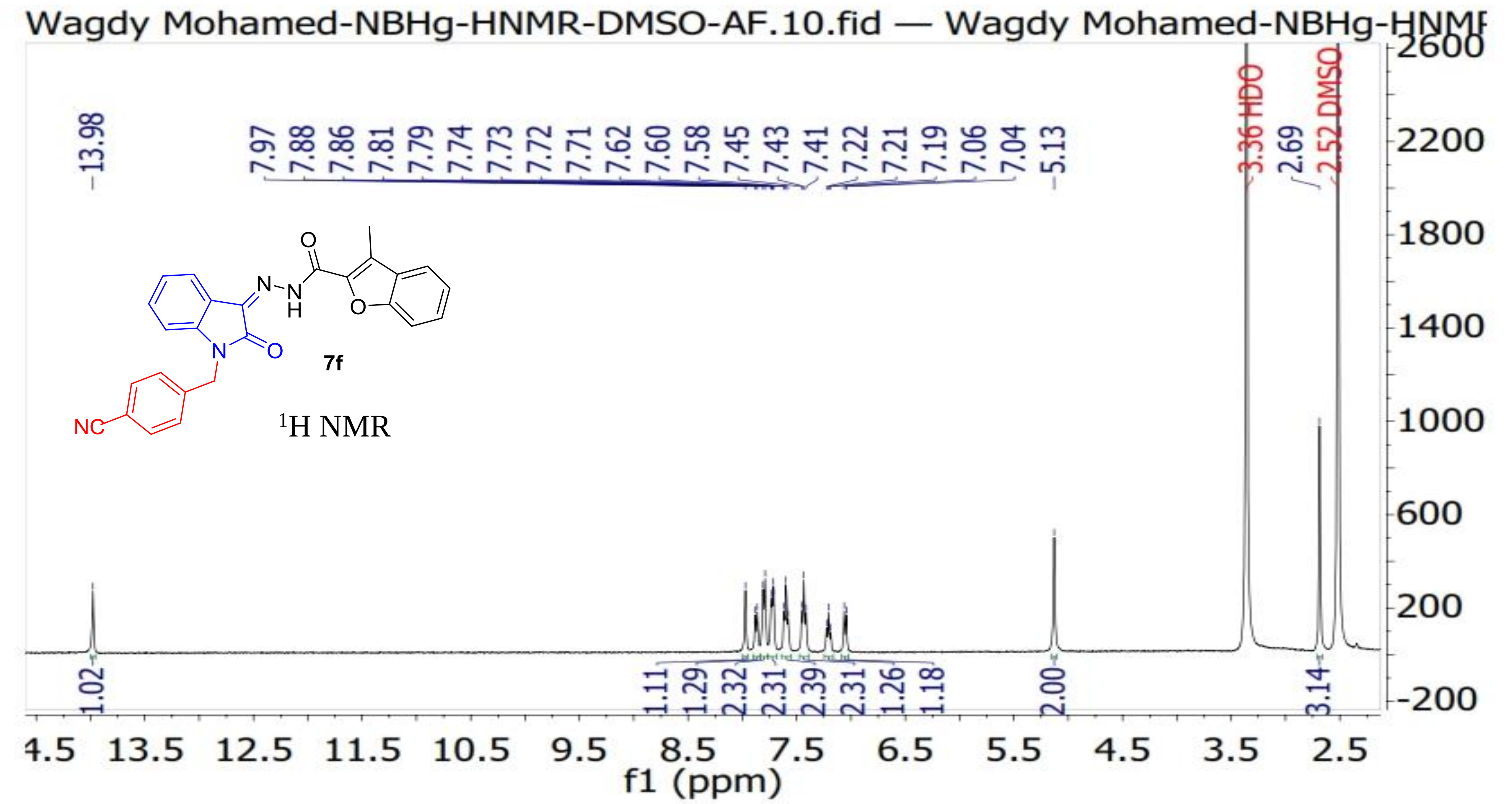
Wagdy Mohamed-NBHg-HNMR-DMSO-AF.10.fid — Wagdy Mohamed-NBHg-HNMR
13.98
7.97 7.88 7.86 7.81 7.79 7.74 7.73 7.72 7.71 7.62 7.60 7.58 7.45 7.43 7.41 7.22 7.21 7.19 7.06 7.04
5.13
3.36 HDO
2.69
2.52 DMSO
7f
1H NMR
1.02
1.11 1.29 2.32 2.31 2.39 2.31 1.26 1.18
2.00
3.14
f1 (ppm)

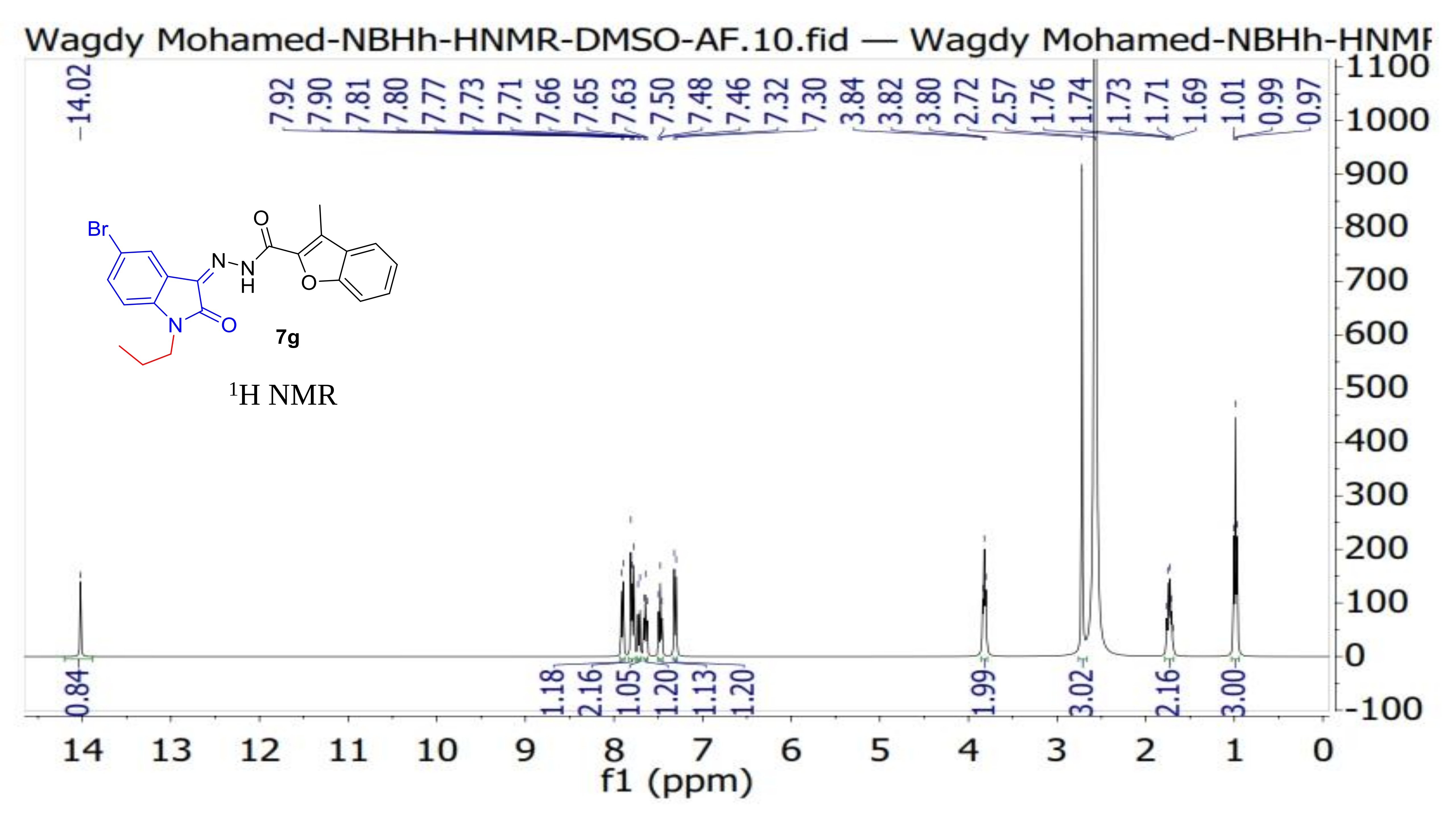
Wagdy Mohamed-NBHh-HNMR-DMSO-AF.10.fid — Wagdy Mohamed-NBHh-HNMF
14.02
7.92 7.90 7.81 7.80 7.77 7.73 7.71 7.66 7.65 7.63 7.50 7.48 7.46 7.32 7.30 3.84 3.82 3.80 2.72 2.57 1.76 1.74 1.73 1.71 1.69 1.01 0.99 0.97
Br
O
N
N
H
O
N
O
7g
1H NMR
0.84
1.18 2.16 1.05 1.20 1.13 1.20
1.99
3.02
2.16
3.00
f1 (ppm)

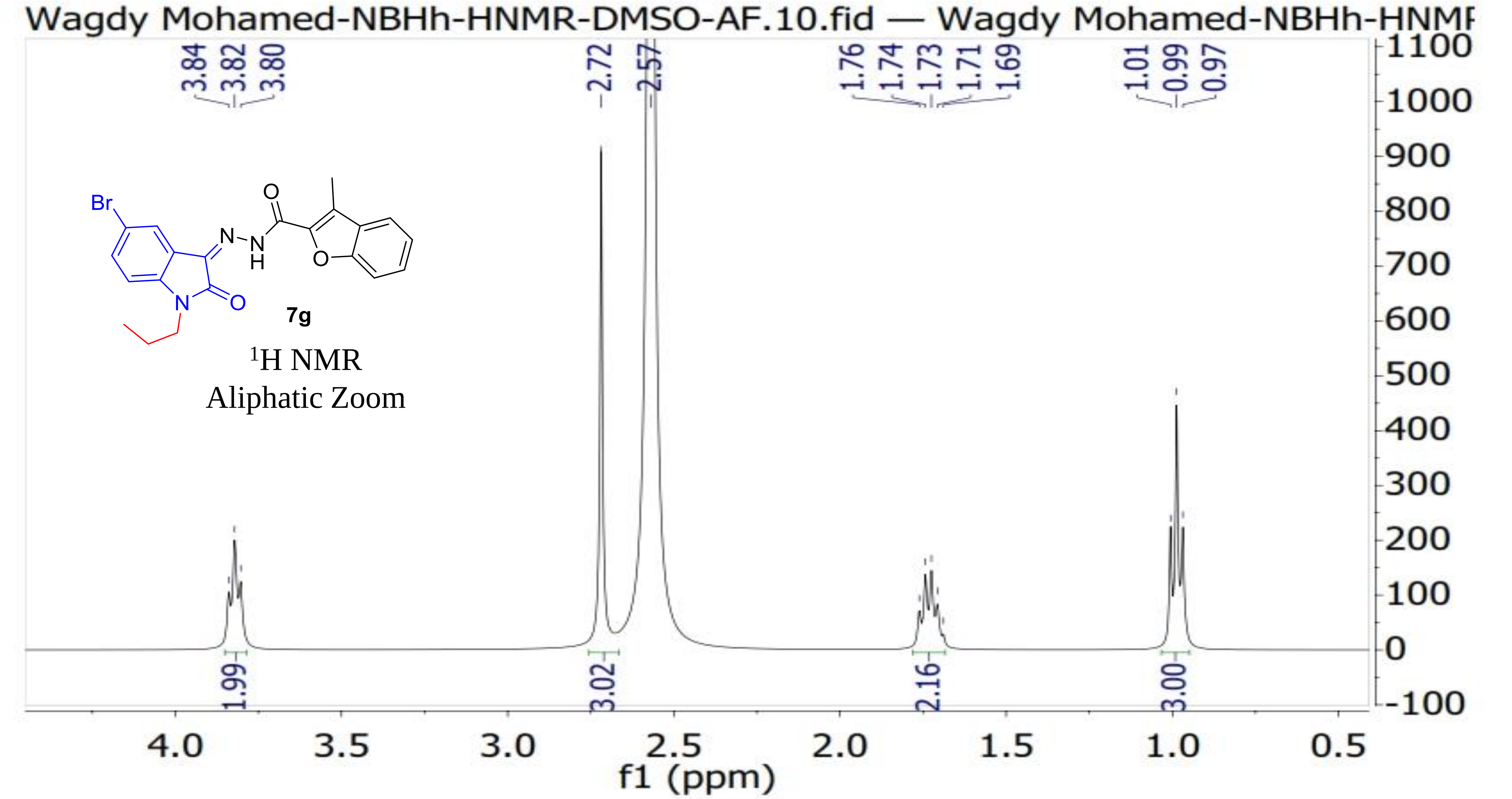
Wagdy Mohamed-NBHh-HNMR-DMSO-AF.10.fid — Wagdy Mohamed-NBHh-HNMR
3.84
3.82
3.80
2.72
2.57
1.76
1.74
1.73
1.71
1.69
1.01
0.99
0.97
Br
N
N
H
O
O
N
O
O
7g
1H NMR
Aliphatic Zoom
1.99
3.02
2.16
3.00
f1 (ppm)

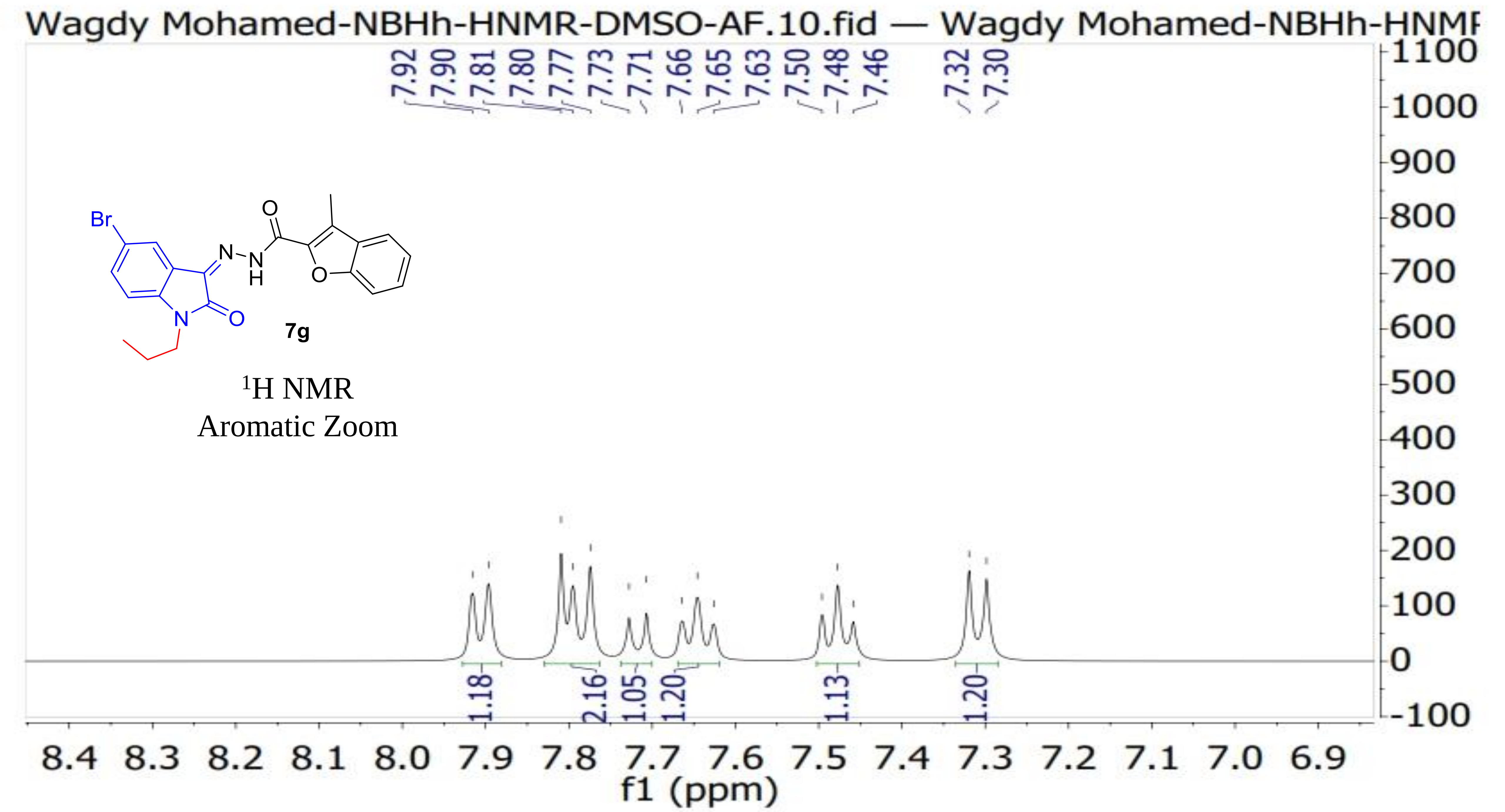
Wagdy Mohamed-NBHh-HNMR-DMSO-AF.10.fid — Wagdy Mohamed-NBHh-HNMR
7.92 7.90 7.81 7.80 7.77 7.73 7.71 7.66 7.65 7.63 7.50 7.48 7.46 7.32 7.30
Br
O
N
N
H
O
N
O
7g
^{1}H NMR
Aromatic Zoom
1.18 2.16 1.05 1.20 1.13 1.20
1100 1000 900 800 700 600 500 400 300 200 100 0 -100
8.4 8.3 8.2 8.1 8.0 7.9 7.8 7.7 7.6 7.5 7.4 7.3 7.2 7.1 7.0 6.9
f1 (ppm)

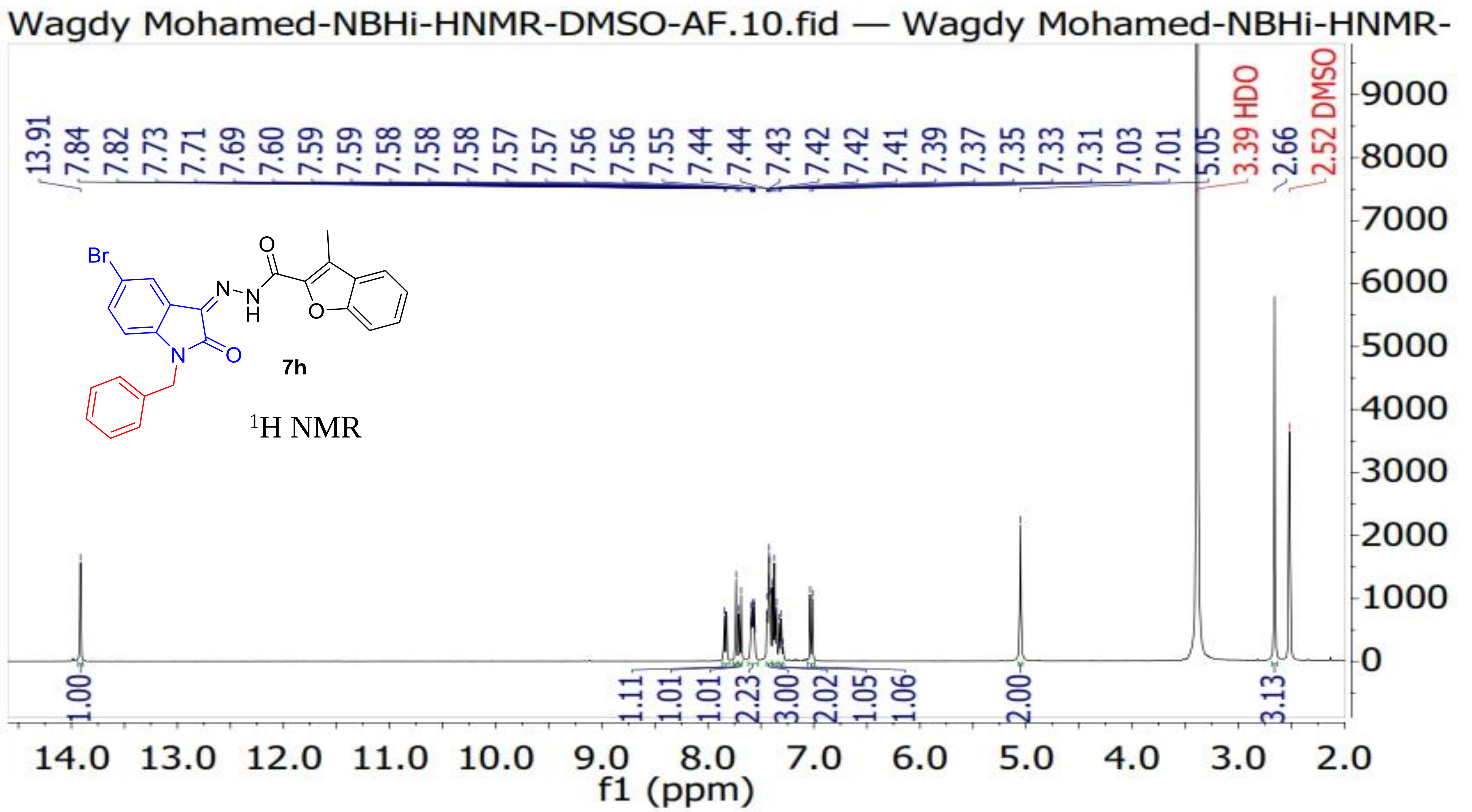
Wagdy Mohamed-NBHi-HNMR-DMSO-AF.10.fid — Wagdy Mohamed-NBHi-HNMR-
13.91
7.84
7.82
7.73
7.71
7.69
7.60
7.59
7.59
7.58
7.58
7.58
7.57
7.57
7.56
7.56
7.55
7.44
7.44
7.43
7.42
7.42
7.41
7.39
7.37
7.35
7.33
7.31
7.03
7.01
5.05
3.39 HDO
2.66
2.52 DMSO
Br
N
N
H
O
O
N
O
7h
1H NMR
1.00
1.11
1.01
1.01
2.23
3.00
2.02
2.05
1.06
2.00
3.13
9000
8000
7000
6000
5000
4000
3000
2000
1000
0
14.0
13.0
12.0
11.0
10.0
9.0
8.0
7.0
6.0
5.0
4.0
3.0
2.0
f1 (ppm)

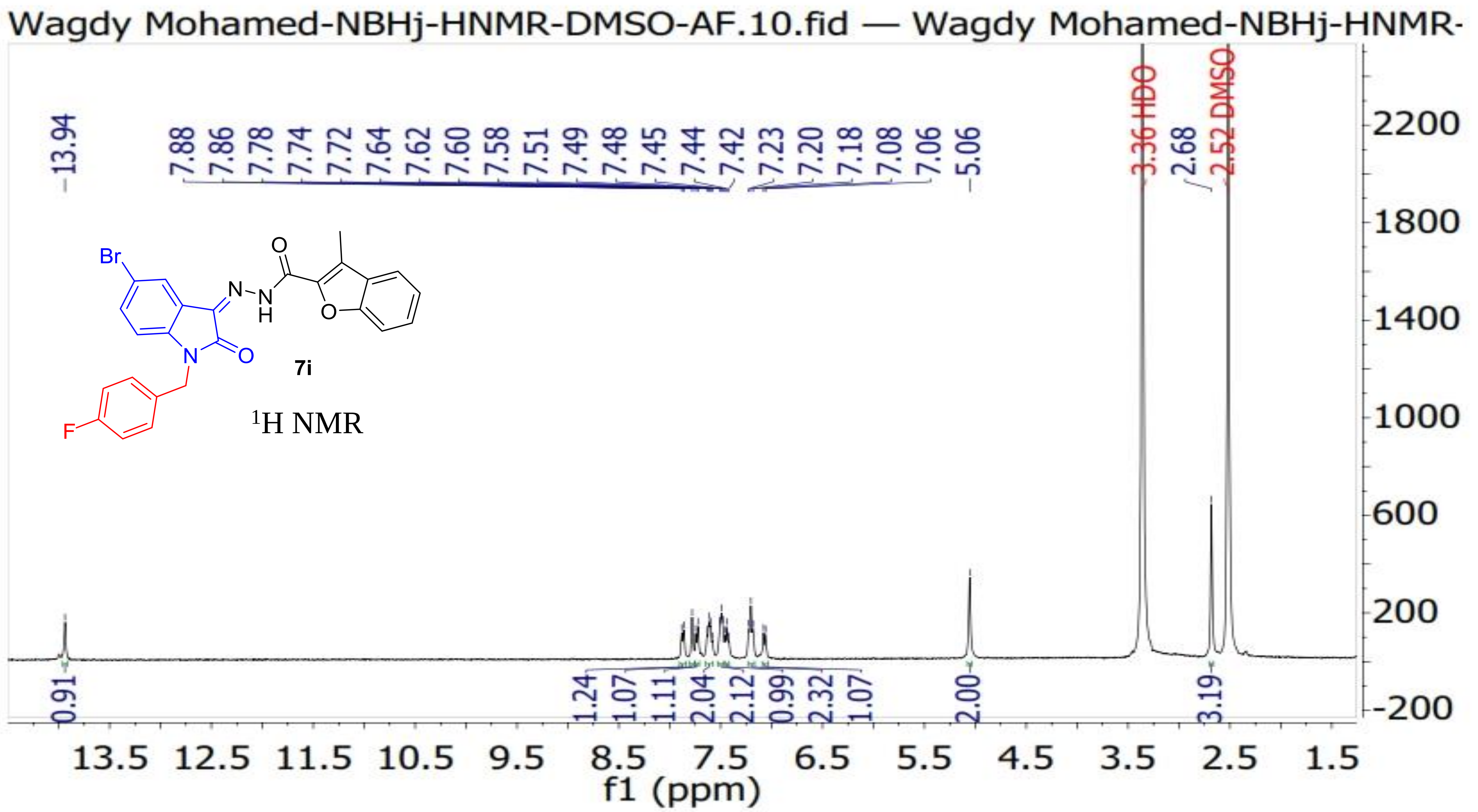

Wagdy Mohamed-NBHj-HNMR-DMSO-AF.10.fid — Wagdy Mohamed-NBHj-HNMR-
13.94
7.88 7.86 7.78 7.74 7.72 7.64 7.62 7.60 7.58 7.51 7.49 7.48 7.45 7.44 7.42 7.23 7.20 7.18 7.08 7.06
5.06
3.36 HDO
2.68
2.52 DMSO
Br
O
N
N
H
O
N
O
F
7i
1H NMR
0.91
1.24 1.07 1.11 2.04 2.12 0.99 2.32 1.07
2.00
3.19
2200
1800
1400
1000
600
200
-200
13.5 12.5 11.5 10.5 9.5 8.5 7.5 6.5 5.5 4.5 3.5 2.5 1.5
f1 (ppm)
